# Functional Polymer Nanocomposite Coatings for Naval and Maritime Applications: Advances in Protection, Durability, and Surface Engineering

**DOI:** 10.3390/polym18192378

**Published:** 2026-09-29

**Authors:** Bianca-Elena Rosca (Neagu), Nicoleta Bogatu, Viorica Ghisman, Daniela Laura Buruiana

**Affiliations:** Interdisciplinary Research Centre in the Field of Eco-Nano Technology and Advance Materials CC-ITI, Faculty of Engineering, “Dunarea de Jos” University of Galati, 47 Domneasca Street, 800008 Galati, Romanianicoleta.simionescu@ugal.ro (N.B.); viorica.ghisman@ugal.ro (V.G.)

**Keywords:** marine corrosion, naval materials, nanocomposite coatings, self-healing coatings, biofouling, sustainable polymers

## Abstract

Marine and naval materials are exposed to complex degradation resulting from the combined effects of chloride-induced corrosion, biofouling, mechanical wear, ultraviolet radiation, and microbiologically influenced corrosion. This review critically examines recent advances in protective coating technologies for improving the durability and sustainability of materials operating in aggressive marine environments. Attention is given to polymer-based and nanocomposite coatings, smart and self-healing systems, sustainable formulations, and advanced deposition and performance-assessment strategies. The reviewed literature shows a clear transition from conventional passive barrier coatings toward multifunctional systems that combine barrier protection with active corrosion inhibition, self-healing, antifouling behavior, and enhanced mechanical resistance. Nanostructured fillers can improve coating performance by restricting the transport of water, oxygen, and chloride ions, while inhibitor-loaded carriers and stimulus-responsive systems provide localized protection after coating damage. Sustainable polymers, bio-based additives, and environmentally compatible inhibitors are also emerging as alternatives to conventional formulations with greater environmental impact. However, long-term durability, scalability, reproducibility, and performance under real marine exposure remain major limitations to industrial implementation. Future progress therefore requires the development of multifunctional and environmentally compatible coating systems supported by standardized multifactor testing, long-term field validation, and scalable industrial processing.

## 1. Introduction

Maintaining the structural integrity and long-term performance of engineering materials used in maritime industries is a central aim in surface engineering and materials science. The expansion of shipping routes, offshore energy systems, and coastal infrastructure has increased the demand for materials capable of operating under severe marine exposure. Naval components, underwater transport systems, port structures, and floating energy platforms are continuously exposed to wave loading, humidity, ultraviolet radiation, temperature fluctuations, and high salinity [1,2]. Seawater is an aggressive electrochemical medium in which dissolved oxygen and chloride ions promote the breakdown of passive films and accelerate material degradation [3]. If these processes are not controlled, they can reduce the mechanical integrity of metallic and concrete structures, leading to cracking, loss of load-bearing capacity, and premature operational failure [4].

In addition to electrochemical degradation, submerged structures are rapidly colonized by marine microorganisms and macrofouling species, which promote biofouling and may further accelerate localized corrosion [5]. For this reason, protective coatings play an important role in extending the operational lifetime of marine and naval infrastructure and in reducing cleaning, repair, and dry-docking requirements [6]. At the same time, concerns associated with toxic biocides, volatile organic solvents, persistence, and ecological accumulation have increased the demand for more environmentally compatible protection strategies [7]. The need for durable protective systems is further supported by long-term exposure studies on structural steels, where exposure to 3.5% NaCl resulted in more pronounced corrosion degradation and localized pitting than exposure to drinking water [8].

Recent developments in polymer science, nanotechnology, and sustainable materials have broadened the range of coating systems available for marine protection. Polymer nanocomposites and other nano-engineered coatings are being investigated to improve barrier and mechanical properties, while bio-based, waterborne, and environmentally compatible formulations aim to reduce the environmental impact associated with conventional coating systems [9,10,11]. These material classes and their protective mechanisms are discussed in detail in Section 3.

In addition to coating composition, surface preparation and deposition conditions strongly influence coating adhesion, morphology, porosity, and long-term performance.

Techniques such as high velocity oxygen fuel (HVOF) spraying and other advanced deposition routes provide different possibilities for controlling coating structure and substrate–coating interactions [11,12]. The main deposition technologies and their influence on coating performance are addressed in Section 4.

This review critically examines protective coating technologies for marine and naval applications by linking degradation mechanisms, coating materials, processing methods, performance evaluation, sustainability, and industrial implementation. Section 2 addresses the principal corrosion and biofouling mechanisms occurring in marine environments. Section 3 examines conventional and advanced protective materials, including polymeric, inorganic, ceramic, metallic, nanocomposite, and sustainable coating systems.

Section 4 discusses coating deposition and surface preparation, while Section 5 focuses on methods used to evaluate coating performance and durability. Economic feasibility, scalability, lifecycle considerations, and industrial perspectives are analyzed in Section 6. Section 7 discusses current limitations and future research priorities, including long-term durability, environmental requirements, multifunctional protection, predictive monitoring, and industrial implementation. Finally, Section 8 summarizes the main conclusions of the review.

### Literature Search and Selection

Relevant scientific literature was identified through web-based academic searches using combinations of topic-specific keywords. The selected publications were subsequently accessed through scientific journal and publisher platforms, including ScienceDirect, SpringerLink, MDPI, and other relevant academic sources. The literature search focused primarily on publications from 2019 to 2026 in order to reflect recent developments in marine protective coatings, nanocomposite systems, smart coatings, sustainable materials, and digital monitoring approaches. Earlier publications were also retained when they provided fundamental concepts, established corrosion mechanisms, methodological principles, or background information necessary for interpreting recent developments.

The literature was identified using individual terms and combinations of keywords such as “marine corrosion”, “naval protective coatings”, “marine polymer coatings”, “nanocomposite coatings”, “epoxy coatings”, “polyurethane coatings”, “zinc-rich coatings”, “thermally sprayed aluminum”, “self-healing coatings”, “marine antifouling coatings”, “microbiologically influenced corrosion”, “electrochemical impedance spectroscopy”, “bio-based coatings”, “green corrosion inhibitors”, “lifecycle assessment”, “predictive maintenance”, “artificial intelligence”, and “digital twin”.

Publications were considered relevant when they addressed coating materials, degradation mechanisms, deposition technologies, performance evaluation methods, environmental sustainability, economic aspects, or industrial implementation related to marine, naval, offshore, or seawater-exposed applications. Priority was given to peer-reviewed research articles and review papers published in English and providing sufficient technical information regarding coating composition, protective mechanisms, processing conditions, testing procedures, or performance.

The selection process was iterative. Titles and abstracts were initially evaluated according to their relevance to the scope of the review, followed by examination of the full text when appropriate. Reference lists of relevant publications were also used to identify additional studies and support literature. Publications with no clear relationship to marine or offshore applications, duplicate sources, and papers providing insufficient technical information for the topics addressed in this review were not prioritized. The literature selection process was therefore designed to ensure broad thematic coverage, scientific relevance, and consistency with the multidisciplinary scope of the review.

## 2. Marine Corrosion in Naval Materials: Mechanisms, Assessment, and Mitigation

The degradation of metallic structures in marine environments is governed by the interaction of electrochemical, environmental, mechanical, and biological factors. Understanding these mechanisms is essential for evaluating the durability of naval and offshore assets and for selecting suitable protective strategies. Marine corrosion is fundamentally an electrochemical process in which anodic metal dissolution is coupled with cathodic reduction reactions involving dissolved oxygen or water [13]. Seawater acts as a conductive electrolyte due to its high salinity and chloride ion content. This increases ionic conductivity, lowers the resistance of the electrochemical circuit, and helps the formation of localized anodic and cathodic regions that sustain metal dissolution [8,14,15].

The main electrochemical reactions involved in steel corrosion in seawater include anodic iron dissolution and cathodic oxygen reduction:

Fe → Fe^2+^ + 2e^−^ (1)

O_2_ + 2H_2_O + 4e^−^ → 4OH^−^ (2)

The corrosion process is commonly characterized by parameters such as corrosion potential (*E_corr_*), corrosion current density (*i_corr_*), and polarization resistance (*R_p_*), which are strongly influenced by chloride concentration, dissolved oxygen, pH, temperature, and electrolyte conductivity.

**Figure 1:**
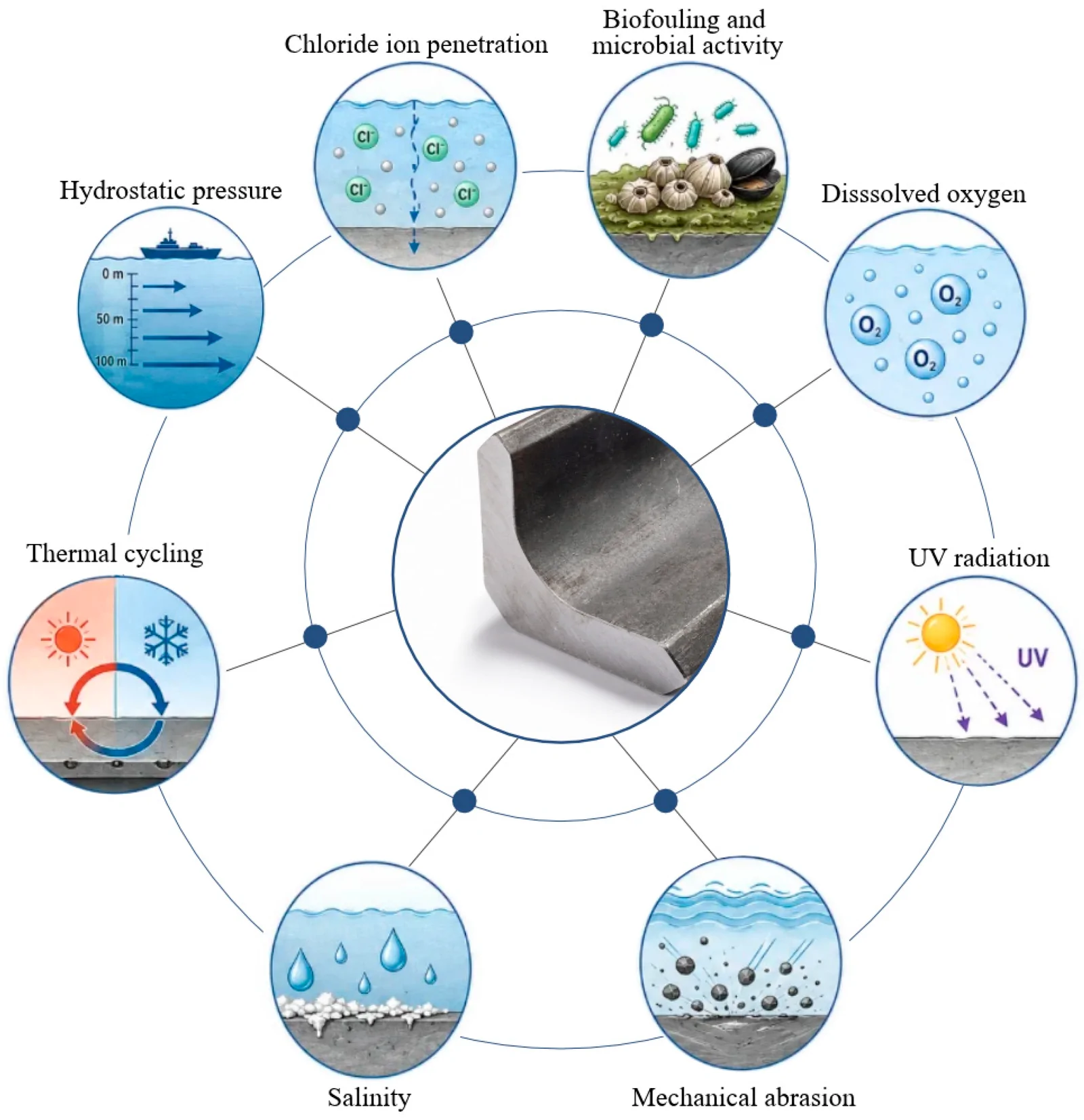
Main degradation factors affecting metallic structures in marine environments.

Figure 1 was prepared by the authors to schematically summarize the main environmental, mechanical, and biological factors contributing to the degradation of metallic structures in marine environments. Salinity and chloride penetration promote electrochemical activity and destabilization of protective surface films, while dissolved oxygen influences the electrochemical corrosion process [3,8,14,15]. UV radiation, temperature variations, and mechanical exposure can further contribute to material and coating degradation under marine service conditions [1,2]. Biofouling and microbial activity represent additional biological factors that may modify the metal–environment interface and promote localized degradation [5].

Among these factors, temperature and dissolved oxygen play a particularly important role in controlling corrosion kinetics and local electrochemical conditions. The rate of metal dissolution is strongly influenced by local temperature variations, which determine the activation energy threshold for the electrochemical reaction. Higher temperatures increase reaction kinetics and can accelerate corrosion processes. Moreover, the presence of dissolved oxygen leads to significant heterogeneity; oxygen saturated surface waters promote cathodic reduction, whereas areas with low oxygen usually located close to the seafloor or in crevices turn into very localized, active anodes [16].

Localized corrosion is particularly critical in marine environments because chloride ions can penetrate and destabilize protective oxide films on steel surfaces [8]. Once the passive layer is locally disrupted, an autocatalytic process may develop. The damaged region becomes an active anodic site, while the surrounding passive surface acts as a cathodic area. This unfavorable cathode-to-anode area ratio accelerates local penetration and can lead to pitting or crevice corrosion before significant uniform mass loss is visible [17,18].

Biological activity adds another layer of complexity to marine corrosion, commonly known as Microbiologically Influenced Corrosion (MIC). Microorganisms, especially sulfate-reducing bacteria (SRB), inhabit structural surfaces, creating thick, varied biofilms. These structures act as biological micro-reactors, creating anaerobic environments that are protected from the surrounding water. Inside these biofilms, the metabolic conversion of sulfates into hydrogen sulfide (H_2_S) creates concentrated, aggressive conditions that can accelerate the degradation of the metallic substrate, reducing the effectiveness of conventional electrochemical protection measures [19,20]. In response to these issues, the development of protective coatings has advanced towards adaptive, “smart” technologies. Instead of depending on fixed, inactive barriers, contemporary systems include effective additives. Biocides with controlled release are embedded in the matrix to inhibit biofilm formation, whereas nanostructured materials create a “labyrinth effect” that obstructs the movement of corrosive agents. Moreover, the incorporation of nanocontainers facilitates self-healing properties; when the coating experiences mechanical damage, these particles initiate a chemical reaction that closes the micro-fissure, maintaining the protective layer without external intervention [21,22]. Ultimately, a deep understanding of these variables ranging from thermal and oxygen driven kinetics to biological metabolic interference is essential for the continued evolution of maritime structural management [23,24].

The understanding of marine corrosion has gradually evolved, moving from an emphasis on static mass-loss assessment to the examination of dynamic, multi-scale interactive systems. Historically, the first comprehension of corrosion was governed by the “barrier paradigm”, which viewed metal deterioration as a deterministic, predictable interaction between a uniform metal surface and a basic electrolyte [25,26].

Nonetheless, the belief in homogeneity turned out to be inadequate when faced with the reality of structural failures in practice. This insight sparked the creation of “localized models,” shifting the emphasis from uniform surface degradation to the destabilization of passive layers. Through the study of the autocatalytic characteristics of pitting, crevice corrosion, and high-cycle corrosion fatigue, researchers started to view the metallic surface not as a smooth plane, but as a complex landscape where small defects could lead to unexpectedly severe localized harm [27,28].

The latest change in this theoretical direction is defined by the incorporation of “biological and adaptive interference.” Contemporary models perceive the marine environment not just as a chemical mixture, but as a multifaceted ecosystem. The incorporation of MIC-where biofilm formations actively adjust the local pH and chemical redox state has necessitated a shift away from abiotic-only forecasts [29,30]. As a result, recent research is increasingly characterized by the move towards adaptive protective strategies.

Current coating design increasingly emphasizes not only durability, but also adaptability and self repair. Advancing toward intelligent coatings and nanostructured additives that support self-repair processes, the field is moving toward protective systems that can interact more actively with the marine environment and improve the long term management of naval engineering assets [31].

### 2.1. Environmental, Mechanical, and Biological Drivers of Marine Corrosion

The structural deterioration of metallic components subjected to marine conditions represents a complex issue, influenced by the interaction between static electrochemical corrosion and repeated mechanical stress. In contrast to terrestrial environments, the metal-electrolyte interface in marine conditions shows considerable non-homogeneity, driven by seasonal changes and fluid dynamics that directly affect corrosion rates. Recent research employing 3D laser scanning has revealed that steel thickness reduction in offshore structures is non-linear, exhibiting pronounced localized differences across various exposure areas splash, tidal, and immersion resulting from differing oxygen levels and mechanical wave influence [32]. This degradation process can be further accelerated by corrosion fatigue, as the interplay between oxidation phenomena and the build-up of small defects can promote crack growth [33]. In this context, improvements in testing methods enable more accurate tracking of crack growth rates, emphasizing that the corrosive environment eliminates the fatigue limit usually seen in dry conditions [34]. Sophisticated predictive models, utilizing Finite Element Analysis (FEA) and stochastic techniques, indicate that the development of localized corrosion, like pitting, should be included in durability evaluations to effectively gauge the lifespan of metal structures. Additionally, advanced crack propagation modeling has shown that the frequency of cyclic loading may influence anodic dissolution rates, highlighting the need for stricter standards in offshore design [35].

In addition to mechanical factors, microbiologically influenced corrosion (MIC) presents an essential biological element. The development of biofilms on metallic surfaces modifies the local electrolyte chemistry, leading to anaerobic micro-environments that enhance the activity of sulfate-reducing bacteria [36]. This phenomenon not only can accelerate the corrosion rate but also diminishes the efficacy of traditional protection techniques, requiring a shift to strategies that incorporate nanotechnology to address microbial colonization [37].

In conclusion, the creation of sustainable corrosion inhibitors, like those derived from hydrazides or imidazole compounds, represents a promising approach to managing the deterioration of metal surfaces [38,39]. These compounds create firm adsorption layers at the metal–electrolyte boundary, following Langmuir adsorption principles, and offer potential alternatives to conventional inhibitors. As a result, the modern comprehension of marine corrosion processes depends on a multi-level strategy, wherein field test data, computational models, and innovative chemical inhibition methods come together to enhance structural integrity in extreme operating conditions [40].

### 2.2. Coupled Ionic-Microbial Corrosion Mechanisms

**Figure 2:**
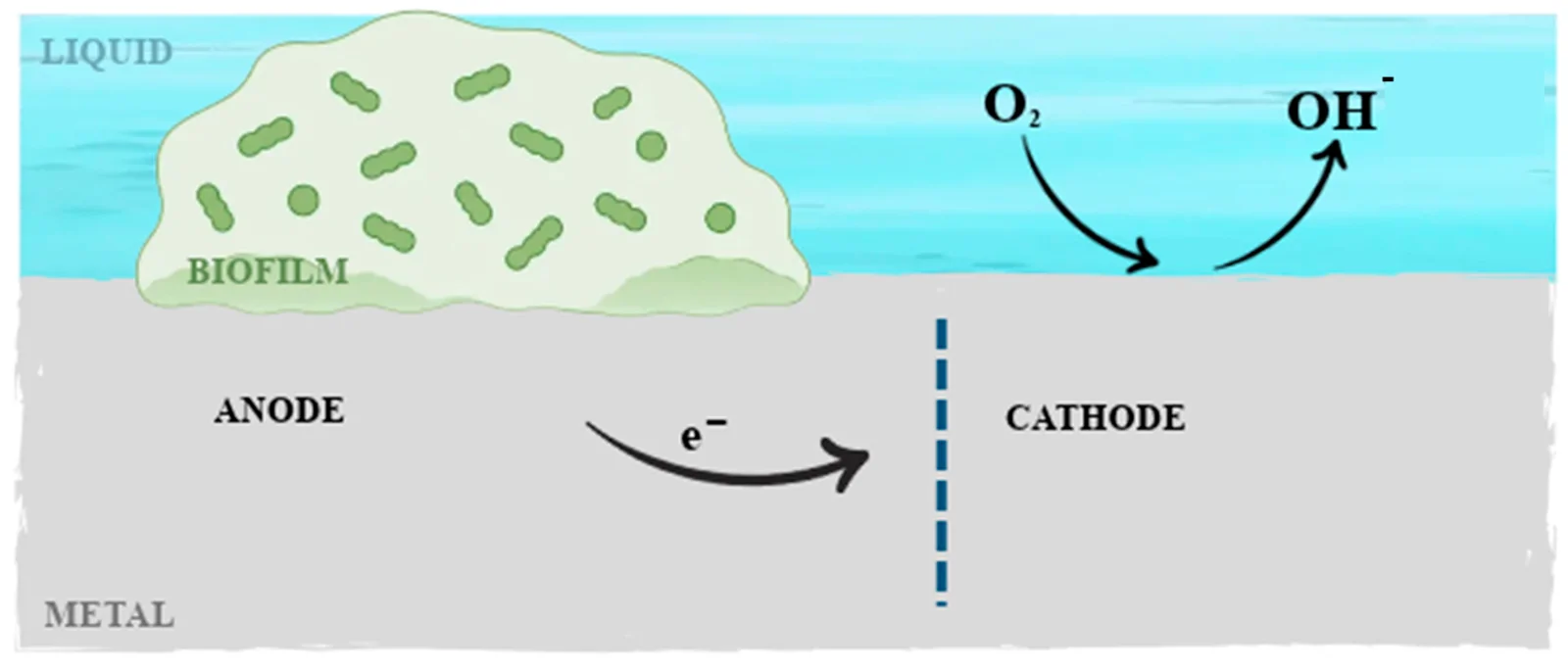
Microbial corrosion mechanisms.

Marine corrosion is not a result of isolated chemical or biological processes, but rather a synergistic interplay where chloride-driven ionic attack and microbial activity mutually enhance degradation rates (Figure 2). The ingress of chloride ions (Cl^−^) is a critical initiator, as these ions disrupt the passive oxide films on structural steels, creating localized anodic sites prone to pitting [41]. This chemical destabilization is highly dependent on environmental zoning; for instance, splash and tidal zones exhibit distinct rust layer stratification and porosity compared to submerged conditions, directly affecting the penetration depth of chloride-induced corrosion [42]. Under actual service conditions, ionic–microbial coupling differs significantly among marine exposure zones. In the splash zone, repeated wetting and drying, strong oxygen renewal, salt accumulation, and wave-induced mechanical action promote coating damage and localized chloride concentration, resulting in highly heterogeneous corrosion patterns [32,41,42]. In the tidal zone, alternating immersion and atmospheric exposure create differential-aeration conditions, while persistent moisture in deposits, surface defects, and sheltered regions can support biofilm development and locally modify pH and redox conditions, thereby increasing susceptibility to localized corrosion [36,41,42]. In continuously submerged and deep-sea environments, reduced oxygen availability, high hydrostatic pressure, low temperature, and limited nutrient transport modify both electrochemical kinetics and microbial activity. Deep-sea microbial communities and biofilms can generate oxygen-depleted microenvironments and corrosive metabolites, including sulfide species, promoting localized corrosion and biomineralization processes [36]. Therefore, the severity and dominant mechanisms of ionic–microbial coupling depend strongly on the specific marine exposure zone and its associated oxygen availability, wetting conditions, hydrodynamics, pressure, and microbial activity.

Once the surface integrity is compromised by ionic attack, the metal substrate provides a receptive environment for microbial colonization. The subsequent microbiologically influenced corrosion (MIC) introduces a biological dimension where bacterial consortia, such as Halomonas titanicae, alter local chemical gradients, including pH and redox potentials [43]. Recent research highlights that this relationship is not unidirectional: chloride-induced surface destabilization can favor microbial attachment, while biofilms and extracellular polymeric substances can modify the local interfacial chemistry, concentrate aggressive species, and create microenvironments that promote MIC and biomineralization processes [44,45]. Furthermore, the anaerobic metabolism of sulfate-reducing bacteria (SRB) within these biofilms produces hydrogen sulfide (H_2_S), creating an aggressive acidic microenvironment that accelerates anodic dissolution beyond what is observed in abiotic conditions [44,46]. In addition to microbial effects, abiotic variables such as chloride ion concentration, temperature, and CO_2_ availability can further intensify corrosion kinetics; experimental work on X65 carbon steel in CO_2_-saturated NaCl solutions showed that increasing chloride concentration and temperature increased the corrosive severity of the environment and influenced the formation of FeCO_3_-based corrosion product layers [47].

MIC involves different microbial groups whose effects depend on their metabolic activity. Sulfate-reducing bacteria (SRB) develop mainly under anaerobic conditions and generate sulfide species, including H_2_S, whereas iron-oxidizing bacteria promote Fe^2+^ oxidation and may contribute to deposit formation and localized electrochemical heterogeneity. Acid-producing bacteria generate acidic metabolites that locally decrease pH and accelerate metal dissolution [36]. Within biofilms, microbial activity and oxygen consumption create heterogeneous pH and oxygen gradients, with oxygen-depleted regions and corrosive metabolites developing near the metal surface [36]. MIC assessment generally combines microbiological and molecular techniques with surface characterization and electrochemical methods, since microbial activity and corrosion damage must be evaluated together [19].

One strategy for mitigating this coupled degradation lies in advanced metallurgical design. The incorporation of alloying elements, particularly copper (Cu), has been investigated as a potential mitigation mechanism. Copper ions released at the metal-electrolyte interface exhibit antimicrobial effects, which may reduce the density of sessile bacterial populations and disrupt the structural integrity of corrosive biofilms [43,48]. As shown by studies on high-copper-bearing steels, this antimicrobial functionality may contribute to improved long-term durability in marine infrastructure, as it effectively interrupts the feedback loop between ionic penetration and biofilm-induced acceleration [48]. Consequently, managing marine corrosion requires a multi-factorial approach that accounts for the coupling of ionic-microbial stressors, moving beyond single-mechanism assessment toward the integration of advanced material resilience and surface bio-inhibition [46,47,48].

### 2.3. From Conventional Inspection to Predictive Corrosion Assessment

This section links marine corrosion mechanisms with the evolution of assessment strategies, showing how conventional inspection methods are increasingly complemented by sensor-based monitoring, machine learning, artificial intelligence, and smart coating systems.

The evolution of corrosion assessment in marine environments reflects a gradual transition from empirical, inspection-based approaches toward predictive, data-driven and materials-integrated monitoring strategies. Traditional methods, including visual inspection, gravimetric measurements, wall-thickness evaluation, ultrasonic testing and periodic maintenance surveys, have long been used to evaluate the degradation state of marine structures [1,3,23]. These techniques remain valuable because they provide direct information on material loss, coating damage, surface condition and structural integrity. However, they are generally discontinuous in time and space, meaning that localized degradation may develop between inspection intervals without being detected at an early stage.

This limitation is particularly relevant for marine corrosion, where damage is often highly localized and influenced by variable environmental conditions. Processes such as pitting corrosion, crevice corrosion, microbiologically influenced corrosion and underfilm corrosion may initiate at small defects, welds, inclusions, coating holidays or areas with restricted oxygen transport. Because these mechanisms do not always produce visible surface damage during their early stages, conventional inspection methods can underestimate the actual risk to structural integrity [16,17,19,36]. In addition, marine infrastructure is often difficult to access, especially on offshore platforms, submerged structures, ballast tanks and deep-water components, where inspection can be costly, time-consuming and dependent on operational or weather conditions.

Weight loss measurements and conventional electrochemical tests provide useful quantitative information, but they also have methodological constraints. Gravimetric analysis can estimate cumulative corrosion loss over a defined exposure period, yet it does not provide continuous information on the initiation or acceleration of localized attack. Similarly, standard polarization methods can estimate corrosion rate and electrochemical behavior, but the interpretation of results may be affected by surface heterogeneity, biofilm formation, corrosion product accumulation and changes in electrolyte chemistry [3,8,16,24]. Therefore, while traditional techniques remain essential for baseline assessment, they are insufficient on their own for complex marine systems where degradation is dynamic, localized and strongly dependent on environmental variability.

Nanostructured materials contribute to modern corrosion assessment and mitigation by improving both barrier performance and coating functionality. Two-dimensional materials, such as graphene-based fillers and other 2D nanomaterials, can increase the tortuosity of diffusion pathways and reduce the transport of water, oxygen, and chloride ions through the coating matrix [49]. When incorporated into organic coatings, these materials can enhance the protective function of the coating while also supporting the development of multifunctional systems with improved mechanical, electrochemical, and environmental stability.

The development of machine learning has further expanded the capabilities of corrosion assessment. Machine learning approaches in corrosion electrochemistry are increasingly used for prediction tasks and corrosion-type identification, because they can process large datasets containing material composition, electrochemical parameters, environmental variables, and exposure conditions [50]. In parallel, intelligent coating systems based on micro- and nanocontainers provide an active material-based response to corrosion initiation. Such systems can store corrosion inhibitors or healing agents and release them when triggered by pH variation, chloride ingress, mechanical damage, or coating delamination [51]. This connection between predictive assessment and active coating response is important because it links early degradation detection with localized corrosion-control mechanisms.

Artificial intelligence has also become relevant for marine corrosion prediction and detection. AI-based approaches can support image-based corrosion recognition, classification of degradation patterns, and interpretation of monitoring data collected from marine structures [52]. More specifically, feature selection and feature engineering have been shown to improve machine learning-based corrosion rate prediction models by transforming raw input variables into descriptors that better capture the influence of alloy composition, environmental exposure, and electrochemical behavior [53]. This is especially relevant for low-alloy steels and other naval materials exposed to marine environments, where corrosion behavior depends on complex interactions between alloy chemistry, chloride concentration, oxygen availability, temperature, and surface condition.

Modern corrosion assessment increasingly relies on continuous monitoring and predictive interpretation. Wireless sensor networks, embedded sensors, and real-time data acquisition systems allow the collection of environmental and electrochemical parameters directly from operating structures [54]. These systems can monitor variables such as humidity, temperature, salinity, pH, coating impedance, strain, moisture ingress, and electrochemical noise. By providing time-resolved information, sensor-based monitoring can detect changes in coating or substrate condition before severe deterioration occurs. This is particularly important for naval and offshore assets, where early detection can reduce maintenance costs and prevent unexpected failures. In offshore wind turbine structures, corrosion monitoring and prognostic approaches are particularly relevant because access for inspection and repair is difficult, costly, and dependent on weather and operating conditions [55].

In parallel with digital monitoring, mitigation strategies have also evolved from passive protection toward active and adaptive coating systems. Conventional epoxy coatings, cathodic protection systems, zinc-rich primers, and other established corrosion-prevention techniques provide important protection but cannot actively respond to localized chemical changes or mechanical damage once the protective layer is compromised [22,25,56]. Smart and nano-hybrid coatings offer a complementary direction by combining passive barrier performance with active mechanisms such as inhibitor release, self-healing, improved adhesion, and enhanced resistance to aggressive species [57]. When combined with sensor-based monitoring, AI-assisted interpretation, and predictive maintenance strategies, these coatings can function not only as protective barriers but also as active components of a broader corrosion-management strategy.

The comparison between traditional and modern corrosion assessment shows that both approaches are still necessary, but their roles are changing. Traditional methods provide essential validation, physical evidence, and standardized reference data, while modern sensor-based, AI-assisted, and smart-coating approaches enable earlier detection, continuous monitoring, and predictive decision-making. For marine and naval structures, the most effective strategy is therefore not the replacement of conventional techniques, but their integration with advanced monitoring systems, nanostructured materials, and adaptive coating technologies. Such an integrated framework can improve the accuracy of degradation assessment, reduce unnecessary maintenance, extend service life, and support safer and more sustainable management of marine assets [49,53,56].

### 2.4. Smart Mitigation Strategies in Marine Corrosion Protection

The operational lifetime of marine infrastructure is increasingly influenced by the capacity to move from conventional, passive protection toward adaptive and multifunctional mitigation strategies. Traditional approaches, including cathodic protection, sacrificial metallic layers, zinc-rich primers and passive barrier coatings, remain essential in marine engineering because they provide reliable and well-established protection against electrochemical degradation [3,25,56]. However, these strategies are often limited when exposed to complex service conditions involving localized chloride accumulation, pH variation, coating defects, mechanical damage, biofilm formation and fluctuating oxygen availability. Under such conditions, passive systems may delay degradation but cannot actively respond to the early stages of localized corrosion or coating breakdown.

Smart mitigation strategies have therefore emerged as a promising direction for extending the service life of naval and offshore materials. These systems are designed not only to isolate the substrate from aggressive species, but also to respond to environmental or damage-related stimuli. Stimulus-responsive coatings can incorporate micro- and nanocontainers loaded with corrosion inhibitors, healing agents or active additives that are released when local conditions change, such as during pH shifts, chloride ingress, mechanical cracking or coating delamination [51,57]. This active response is particularly relevant for marine structures, where small coating defects can rapidly become initiation sites for pitting corrosion, underfilm corrosion or microbiologically influenced corrosion.

A key advantage of smart coatings is their ability to combine passive and active protection within the same architecture. The polymer matrix provides the initial physical barrier against water, oxygen and chloride ions, while embedded functional carriers supply a localized protective response when the barrier is damaged. Inhibitor-loaded nanocontainers, halloysite nanotubes, layered double hydroxides, mesoporous particles and polymeric capsules can release active compounds at defect sites, reducing anodic dissolution and cathodic reactions before corrosion propagates extensively [51,57]. This mechanism is particularly valuable for naval components that are difficult to inspect or repair frequently, such as ballast tanks, submerged structures, offshore foundations and internal seawater systems.

The incorporation of two-dimensional nanomaterials, including graphene-based materials and MXenes, further improves smart mitigation strategies by increasing the tortuosity of diffusion pathways within the coating. These materials can reduce the transport rate of aggressive species toward the metal substrate, while also improving mechanical reinforcement and barrier stability [49,58]. When combined with self-healing or inhibitor-release mechanisms, nanostructured coatings can provide a more durable protection system than conventional polymer matrices alone. However, their effectiveness depends strongly on filler dispersion, interfacial compatibility, loading concentration and long-term stability in saline environments. Experimental work on coated structural steel exposed to marine conditions further confirms the importance of coating architecture, showing that multilayer systems based on zinc-rich epoxy primer, polyurethane topcoat and functional mineral particles can improve the corrosion protection of steel in seawater exposure [59].

Sustainable corrosion mitigation is another important component of future smart protection systems. Due to increasing environmental restrictions, the use of toxic chromates, heavy metals and persistent synthetic inhibitors is being progressively reduced. As a result, research has shifted toward green corrosion inhibitors derived from plant extracts, natural polymers, amino acids, selected low-toxicity ionic liquids and eco-friendly nanoparticles [40,60,61]. These compounds can adsorb onto metallic surfaces and form protective films that reduce charge transfer and limit corrosive attack. Their integration into coating matrices or nanocontainers offers the possibility of combining environmental compatibility with active corrosion control.

The main challenge is to ensure that these smart and green systems remain effective under real marine exposure. Laboratory studies often demonstrate high inhibition efficiency or self-healing capacity under controlled conditions, but field environments involve variable salinity, temperature, UV radiation, hydrodynamic forces, microbial activity and mechanical abrasion. These factors can influence inhibitor release, coating adhesion, nanofiller stability and healing efficiency. Therefore, future research should focus on long-term validation, cyclic damage testing, real seawater exposure and compatibility with industrial coating application methods. Smart mitigation strategies represent a major step toward a lifecycle-oriented approach to marine corrosion protection. By integrating passive barrier effects, active inhibitor release, nanostructured reinforcement, self-healing behavior and environmentally safer additives, these systems can reduce maintenance frequency and improve the reliability of naval materials. Rather than replacing conventional protection methods entirely, smart coatings should be viewed as complementary technologies that enhance existing systems and enable more durable, sustainable and adaptive protection in aggressive marine environments [49,55,61].

## 3. Materials for Marine Corrosion Protection

The structural integrity of marine infrastructure is fundamentally challenged by the relentless electrochemical degradation induced by harsh, electrolyte-rich environments. For decades, the maritime industry has relied on traditional barrier coatings to mitigate these risks; however, the escalating demand for increased service life, reduced maintenance intervals, and stringent environmental regulations regarding volatile organic compounds (VOCs) has encouraged the development of more durable and environmentally compatible coating materials [6]. This chapter examines the transition from conventional, passive protective layers toward advanced, multifunctional material architectures.

The current landscape of marine corrosion protection is defined by a multi-criteria optimization process, where mechanical robustness, chemical inertia, and environmental compatibility are no longer mutually exclusive requirements. As highlighted by recent advancements in high-performance polymers, including the emergence of non-isocyanate polyurethanes (NIPUs) as sustainable alternatives to traditional precursors, the field is evolving toward coating formulations with improved safety and reduced environmental impact [62]. In parallel, graphene-based materials have attracted considerable interest in marine corrosion protection because their two-dimensional structure can increase diffusion tortuosity and reduce the transport of aggressive species through polymeric coatings [63].

A critical focus of this chapter is the exploration of active and adaptive protective mechanisms. Smart self-healing anticorrosion coatings are increasingly investigated because they can respond to coating damage through inhibitor release, microcrack repair, or restoration of local barrier properties [64]. At the same time, the mechanical behavior of protective coatings against marine fouling stays important, since coating films must resist not only electrochemical attack but also cleaning operations, abrasion, biological attachment, and mechanical wear [65]. For offshore structures, underwater in situ repairing coatings further illustrate the practical importance of curing behavior, adhesion, and repairability under wet or submerged service conditions [66].

**Figure 3:**
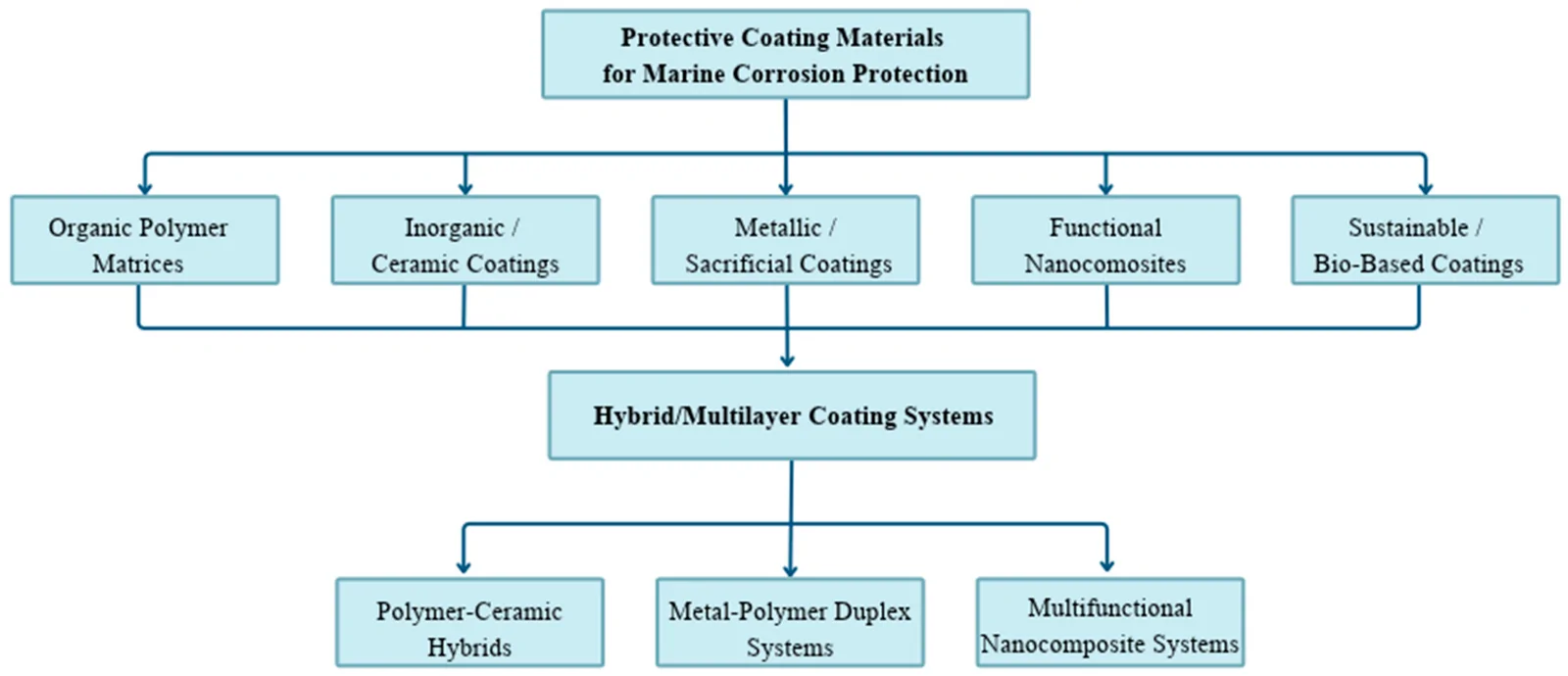
Classification of protective coating materials for marine corrosion protection.

The functionality of polymer-based coating systems can also be improved through the incorporation of fillers and reinforcing agents. Fillers added to epoxy resins can modify mechanical strength, toughness, barrier properties, and resistance to environmental degradation, depending on their dispersion, interfacial compatibility, and loading concentration [67]. The classification of protective coating materials for marine corrosion protection is presented in Figure 3.

These categories are not mutually exclusive, since modern marine coating systems increasingly combine polymeric, inorganic, metallic, and nanostructured components in hybrid or multilayer architectures to integrate barrier, sacrificial, mechanical, and active protection mechanisms.

Comparative degradation studies on zinc-rich epoxy primers combined with acrylic polyurethane topcoats also show that water and ion permeation strongly influence coating failure under simulated marine-industrial atmospheric conditions [68]. Moreover, graphene-based polymer coatings have been widely reviewed as promising systems for preventing marine corrosion, particularly because graphene derivatives can improve barrier performance, mechanical reinforcement, and coating durability when properly dispersed within the polymer matrix [69].

### 3.1. Organic Polymer Matrices

Synthetic polymers stand for one of the main protection strategies against marine-induced electrochemical degradation, serving as a principal component of contemporary protective coating systems. Their utility in marine environments is fundamentally derived from their capacity to form a barrier that isolates metallic substrates from the combined ingress of moisture, chloride ions, and dissolved oxygen, the key catalysts of corrosion. Protective polymeric films, including conducting polymers and polymer composites/nanocomposites, have therefore been widely investigated for industrial substrates exposed to aggressive environments [70].

Epoxy resins represent the most prevalent class of organic matrices commonly used in marine infrastructure, valued for their high cross-linking density and chemical stability. Once cured, the three-dimensional network of the epoxy matrix can provide high adhesion strength to metallic surfaces, often ranging from 30 to 35 MPa, which contributes to coating integrity even under hydrostatic pressure [66,67]. Despite these advantages, the inherent brittleness of highly cross-linked epoxy systems remains a critical limitation in dynamic maritime environments. To address this, current research increasingly focuses on the integration of specific fillers and reinforcing agents that modulate fracture toughness without compromising the barrier properties [65,67]. In this context, recent nanotechnological advancements in polymer nanocomposite coatings are particularly relevant, as they can further enhance anticorrosion, antifouling, and self-healing performance in epoxy-based protective systems [71].

In contrast, polyurethane (PU) coatings offer a distinct mechanical profile characterized by high elasticity and energy dissipation capacity [59]. With elongation at break values frequently reaching 50% to 100%, polyurethane matrices are well suited for structures subjected to periodic mechanical stresses, such as deck coatings and ice-going vessels [66,72]. Their ability to accommodate the expansion and contraction cycles induced by thermal fluctuations ensures that the protective film maintains its continuity over prolonged service periods. Furthermore, the chemical versatility of polyurethanes allows for the synthesis of advanced non-isocyanate derivatives (NIPUs), which align with the emerging industrial requirement for sustainable, low-toxicity coating formulations [62]. This direction is also consistent with current research on environmentally friendly antifouling coatings, which aims to reduce ecological impact while maintaining protective performance in marine settings [73].

The performance of these polymer matrices is significantly influenced by the exposure conditions. Comparative studies of coating degradation specifically those involving zinc-rich epoxy primers paired with acrylic polyurethane topcoats demonstrate that the permeation rate of water and aggressive ions is the primary determinant of system failure, with degradation rates accelerating notably in simulated marine-industrial atmospheres [68]. Consequently, the strategic selection of organic matrices necessitates a data-driven approach that balances the rigid, high-adhesion characteristics of epoxy resins with the flexible, abrasion-resistant properties of polyurethanes. Future advancements, particularly those exploring the incorporation of nanostructured fillers and intrinsic self-healing mechanisms, aim to address the limitations of current organic matrices, supporting the development of more durable marine coating systems [64,71,74].

### 3.2. Inorganic and Ceramic Coating Systems

Inorganic and ceramic-based coatings constitute a pivotal class of materials for protecting marine infrastructure, particularly where operational conditions involve extreme temperatures and high mechanical loads [73,74]. Unlike organic polymer matrices, which are prone to thermal softening and photodegradation under intense UV exposure, ceramic systems can provide high chemical stability and oxidation resistance. These coatings function as robust, non-metallic barriers that maintain their structural integrity even when subjected to the harsh, electrolyte-rich conditions typical of maritime operations [73].

An important development in this field is the use of phosphate-based ceramic coatings modified with passivated aluminum particles to mitigate corrosion in high-temperature marine atmospheres. Research has shown that the incorporation of passivated aluminum creates a synergistic protective effect; the aluminum particles serve as sacrificial elements that reinforce the ceramic matrix, while simultaneously promoting the formation of a dense, passive film on the metallic substrate [75]. This dual mechanism enables the coating to withstand multiple cycles of salt spray and high-temperature stress without compromising its protective performance, an improvement over monolithic ceramic layers under the tested conditions [75].

Furthermore, the integration of nanostructured inorganic fillers has contributed to improved anticorrosive coating performance. For instance, the use of silicon carbide (SiC) nanoparticles functionalized with graphene oxide (GO) within an epoxy matrix has been shown to effectively address the common issue of nanoparticle aggregation [76]. This approach enhances the mechanical hardness and abrasion resistance-key attributes for components like propellers or pumps exposed to high-velocity seawater-while simultaneously creating a tortuous path for water molecules, thereby reducing the rate of electrolyte ingress [76]. The hybridization of these inorganic reinforcements with organic matrices provides a sophisticated solution that leverages the intrinsic hardness of ceramics alongside the flexibility of polymers [67,76].

The suitability of these materials for high-temperature marine applications is often determined by the thermal stability of the system, which evaluates the ability of the coating to retain chemical and mechanical integrity under elevated temperatures and thermal cycling. As demonstrated in various marine material studies, the selection of ceramic architectures-potentially incorporating intelligent, self-healing nanocontainers-promises to further extend the operational life of critical marine assets, moving the industry toward more durable and proactive corrosion mitigation strategies [71,74,77].

### 3.3. Metallic and Sacrificial Coating Systems

Metallic and sacrificial coatings represent one of the most established strategies for the active mitigation of electrochemical degradation in marine engineering, functioning primarily based on the principle of cathodic protection via sacrificial anodes. Among these systems, thermally sprayed aluminum (TSA) coatings have been widely investigated for marine environments because they can provide sacrificial protection to steel substrates exposed to seawater and offshore service conditions [78]. By applying metals with a more negative electrode potential than steel, such as zinc or aluminum, to the substrate, a galvanic cell is generated in which the coating layer undergoes preferential oxidation, thereby protecting the underlying steel from anodic dissolution in chloride-containing environments.

Despite their reported electrochemical performance, a critical limitation of pure metallic layers, such as TSA, lies in their low resistance to mechanical stress and wear; a significant portion of damage occurs even during transport and assembly operations. In dynamic applications, such as mooring lines or elements in the splash zone, abrasive stresses occurring simultaneously with corrosive attacks lead to accelerated degradation via tribocorrosion [79]. To overcome this barrier, innovative duplex systems have been investigated by modifying the TSA surface using the Plasma Electrolytic Oxidation (PEO) technique at high voltages of 300–600 V [79]. This process partially converts the outer aluminum layer into a hard ceramic alumina film, increasing surface layer hardness by up to 25 times and reducing synergistic degradation through abrasion and delamination fatigue in seawater [79].

The efficiency and service life of metallic sacrificial coatings are directly dictated by their metallurgical properties, microstructure, porosity, sealing quality, and the deposition technology selected in relation to the specific marine exposure zone. For this reason, the prediction of thermal spray coating performance increasingly relies on the combined use of laboratory tests and field exposure data, which allows a more realistic evaluation of long-term behavior in marine environments [80]. Established industrial processes include hot-dip galvanizing and thermal spraying. While hot-dip galvanizing can achieve strong metallurgical adhesion through the diffusion of zinc into the steel, generating complex intermetallic phases, TSA offers geometric versatility for large-scale offshore structures and marine offshore wind turbine structures, and has been reported to provide long-term protection when properly sealed [78,80].

A major technological evolution in the field of galvanic coatings is the shift from classic pure zinc layers to advanced ternary zinc-magnesium-aluminum (Zn-Mg-Al) alloys. Recent experimental research has demonstrated that the addition of magnesium and aluminum to the molten zinc bath induces fine grain refinement and promotes the formation of stable and relatively insoluble corrosion products under marine atmospheric conditions [81]. These products include zinc hydroxychloride, aluminum oxyhydroxide, and layered double hydroxides [81]. This dense film of secondary products acts as an effective passivation barrier, slowing the kinetics of anodic zinc dissolution and extending the service life of structural steel.

Recent research on advanced zinc–polymer composites has further shown that zinc-based sacrificial protection can be combined with polymeric matrices and self-healing functionality, leading to hybrid coating systems that integrate galvanic activity, barrier resistance, and damage-responsive behavior for marine corrosion protection [82].

More broadly, surface protection technologies for metallic materials in marine environments increasingly combine metallic, ceramic, polymeric, and hybrid strategies depending on the targeted degradation mechanism and exposure conditions [83]. In this context, protective polymeric coatings can complement sacrificial metallic layers by improving barrier resistance, reducing electrolyte transport, and enhancing long-term coating stability in marine applications [84]. Thus, modern metallic systems are evolving from simple galvanic barriers into hybrid and adaptive protective layers with potential to improve the long-term durability of critical marine infrastructure.

### 3.4. Functional Nanocomposites

The integration of nanotechnology into anticorrosive coating systems has become an important research direction for improving the protection of marine infrastructure [59]. Nanocomposite coatings are defined as hybrid systems in which nanostructured fillers are dispersed within a polymer matrix to enhance the physical, chemical, mechanical, and electrochemical resistance of the coating [85]. The incorporation of nanoparticles not only improves the barrier effect but also imparts novel functionalities such as anti-fouling behavior, photocatalytic activity, and autonomous self-healing properties.

In marine applications, antifouling nanocomposite polymer coatings are particularly relevant because they can combine corrosion protection with the reduction of biological attachment on exposed surfaces [86]. These capabilities are valuable in marine environments, where continuous exposure to saltwater, UV radiation, and bio-colonization can accelerate coating degradation and reduce long-term protective performance [84,86,87]. By integrating functional nanofillers into polymer matrices, nanocomposite coatings can provide enhanced barrier properties, improved surface stability, and additional functionalities such as antifouling behavior, self-healing response, and reduced permeability to aggressive species [86].

A defining feature of nanocomposite coatings is the high surface-area-to-volume ratio of the nanofillers, which facilitates strong interfacial interactions with the polymer matrix and promotes uniform particle dispersion. This uniformity reduces the formation of micro-defects and porosity, effectively increasing the “tortuosity” of the diffusion pathways for corrosive agents [85]. By forcing water molecules, oxygen, and chloride ions to navigate around dispersed nanoparticles, the effective diffusion coefficient may be reduced. The incorporation of different nanoparticles has also been reported to improve impact resistance and water barrier properties, which are important for coating durability under marine exposure [88].

Among the most studied nanomaterials, titanium dioxide (TiO_2_) and zinc oxide (ZnO) have received considerable attention for multifunctional coating performance. Nano-TiO_2_ can contribute to corrosion protection through barrier enhancement and, under controlled conditions, photocatalytic activity. Under UV illumination, TiO_2_ can generate reactive oxygen species (ROS), which may degrade organic contaminants on the coating surface and reduce biofilm development, thereby limiting the risk of microbiologically influenced corrosion (MIC) [86,89]. However, this functionality must be carefully controlled, since excessive photocatalytic activity may also affect the long-term stability of polymer matrices. In addition, studies indicate that the inclusion of metal oxide nanoparticles, such as SiO_2_, TiO_2_, and Al_2_O_3_, into epoxy matrices has been reported to improve surface hardness and water barrier properties, limiting swelling and delamination during prolonged seawater exposure [79,88].

The recent development of nanocomposite coatings has introduced additional intelligent functions. Self-healing nanocomposites, often based on halloysite nanotubes or metal–organic frameworks loaded with corrosion inhibitors, can contribute to the repair of microcracks and the suppression of localized electrochemical activity at damaged sites [82,85]. When coating damage occurs, these nanocarriers may release active compounds that either inhibit anodic and cathodic reactions or help restore the protective function of the coating. These multifunctional systems indicate an important direction in corrosion protection, where responsive and adaptive mechanisms can complement conventional passive barrier coatings rather than fully replace them.

Despite these advantages, nanocomposite coating performance is highly sensitive to nanofiller dispersion and filler–matrix interfacial compatibility. Agglomerated nanoparticles can generate local heterogeneities, microdefects, and preferential pathways for water and chloride transport, thereby reducing rather than improving barrier performance.

Similarly, weak interfacial bonding between the nanofiller and polymer matrix may promote local debonding and reduce mechanical reinforcement. Consequently, nanofiller concentration, dispersion method, and surface functionalization must be carefully controlled to obtain reproducible improvements in marine coating performance [76,85,88].

### 3.5. Sustainable and Bio-Based Coating Solutions

The development of sustainable and bio-based coatings is driven by stricter environmental regulations and by the need to reduce the use of volatile organic compounds, hazardous air pollutants, and toxic marine biocides. In marine applications, this transition is particularly important because coating degradation, leaching, and antifouling additives can directly affect aquatic ecosystems. Recent developments in titanium dioxide-based materials for aquatic antifouling applications show that photocatalytic and environmentally oriented surface strategies are increasingly explored as alternatives to conventional toxic antifouling systems [89]. At the same time, systematic reviews of corrosion inhibitors in marine environments highlight the growing interest in less hazardous inhibitor systems capable of limiting corrosion while reducing environmental impact [90]. Bio-based polymeric coatings are therefore being investigated as sustainable and multifunctional solutions for corrosion and mechanical protection [91]. Life cycle assessment studies further emphasize that the environmental impact of anticorrosion coating systems depends not only on their chemical composition, but also on raw material origin, application method, durability, maintenance frequency, and end-of-life treatment [92].

Bio-based polymers derived from renewable resources, including cellulose, lignin, chitosan, and various plant-derived resins, offer promising alternatives to petrochemical-based systems. Cellulose derivatives are particularly relevant because their abundance, biodegradability, and functional groups support their use in environmentally oriented materials and contaminant-control applications in aquatic environments [93]. In marine coatings, the combination of bio-based polymers with natural antifoulants has also been investigated as an eco-friendly strategy for reducing biological attachment while maintaining coating functionality [94]. More broadly, eco-friendly polymer composites derived from renewable or biodegradable resources provide a useful material platform for reducing dependence on conventional petroleum-based polymers [95].

Recent research highlights that lignin, a polyphenolic byproduct of the pulp and paper industry, can be utilized as a bio-additive in epoxy resins to provide intrinsic UV-shielding and antioxidant properties [96]. When acetylated, lignin has been reported to reduce photodegradation rates and surface oxidation, as confirmed by FTIR and XPS analyses, offering a coal tar-free alternative for maritime corrosion protection [96]. In addition, recent reviews of polymer-based anticorrosion coatings emphasize that sustainable coating development must balance barrier performance, adhesion, mechanical durability, environmental stability, and compatibility with functional additives [97].

The integration of green corrosion inhibitors represents an important research direction in sustainable chemistry. Natural extracts, such as those from Equisetum arvense or various plant tannins, are being explored for their ability to adsorb onto metallic surfaces and form protective films, potentially suppressing localized corrosion without the toxicity associated with chromate-based primers [98]. These bio-derived molecules may be incorporated into coating matrices or compatible carrier systems to support controlled release, long-term protection, and reduced environmental loading, although their stability and effectiveness under real marine exposure still require further validation.

The mechanical and barrier integrity of these systems can be further strengthened by nanostructured reinforcement. Cellulose nanocrystals (CNCs) and other biomass-derived nanofibers are increasingly used to create tortuous diffusion paths within waterborne coating matrices. By enhancing hydrogen bonding and physical entanglement, these bio-fillers can improve water resistance and tensile strength, contributing to the development of more durable environmentally compatible coating systems [93,97]. Moreover, life cycle assessment studies demonstrate that bio-based coatings may reduce the environmental footprint, depending on formulation, durability, and lifecycle boundaries [92].

From a lifecycle perspective, the environmental benefit of a bio-based coating cannot be assessed solely from the renewable origin of its raw materials. Life cycle assessment should consider raw-material extraction, synthesis and formulation, coating application, energy and solvent consumption, maintenance frequency, service-life extension, and end-of-life treatment. A formulation with a lower initial environmental burden may not provide an advantage if its marine durability is insufficient and frequent recoating is required [92]. Therefore, long-term degradation behavior is a critical sustainability parameter. Water uptake, hydrolysis, swelling, ultraviolet exposure, mechanical damage, and biological activity may progressively modify the polymer network, reduce adhesion, and increase permeability to aggressive species. For bio-based systems, biodegradability must consequently be balanced against the requirement for controlled and sufficiently long service life in marine environments [91,92,97].

Ecotoxicity is another important consideration because environmentally oriented formulations may still release additives, degradation products, corrosion inhibitors, or nanoscale components into seawater. Consequently, renewable or naturally derived origin should not automatically be considered equivalent to environmental safety. The toxicity, persistence, leaching behavior, and possible effects of released compounds on aquatic organisms should be evaluated together with coating performance [89,90,92,94]. From a regulatory perspective, the progressive restriction of hazardous biocides, volatile organic compounds, and persistent toxic additives encourages the development of waterborne, low-VOC, bio-based, and non-toxic antifouling systems. However, future marine coatings must demonstrate both protective efficiency and acceptable environmental behavior throughout their lifecycle, including application, service, degradation, and disposal stages [89,90,92].

In conclusion, the convergence of sustainable chemistry and advanced nanotechnology is reshaping marine infrastructure protection. As research into biopolymer chemistry, natural antifoulants, and green additives progresses, bio-based coatings are showing potential for broader industrial adoption, with the aim of balancing performance requirements with environmental responsibility [91,94,97].

A comparative overview of the main coating classes discussed in this section is provided in Table 1, highlighting their representative performance, principal advantages and limitations, and relevance for marine applications.

## 4. Deposition Techniques for Marine Protective Coatings

The performance of marine protective coatings depends strongly on the deposition method, surface preparation, and interfacial quality. In addition to coating formulation, parameters such as particle velocity, substrate temperature, layer thickness, porosity, curing conditions, residual stress, electrolyte chemistry, and post-treatment conditions influence long-term electrochemical stability.

**Figure 4:**
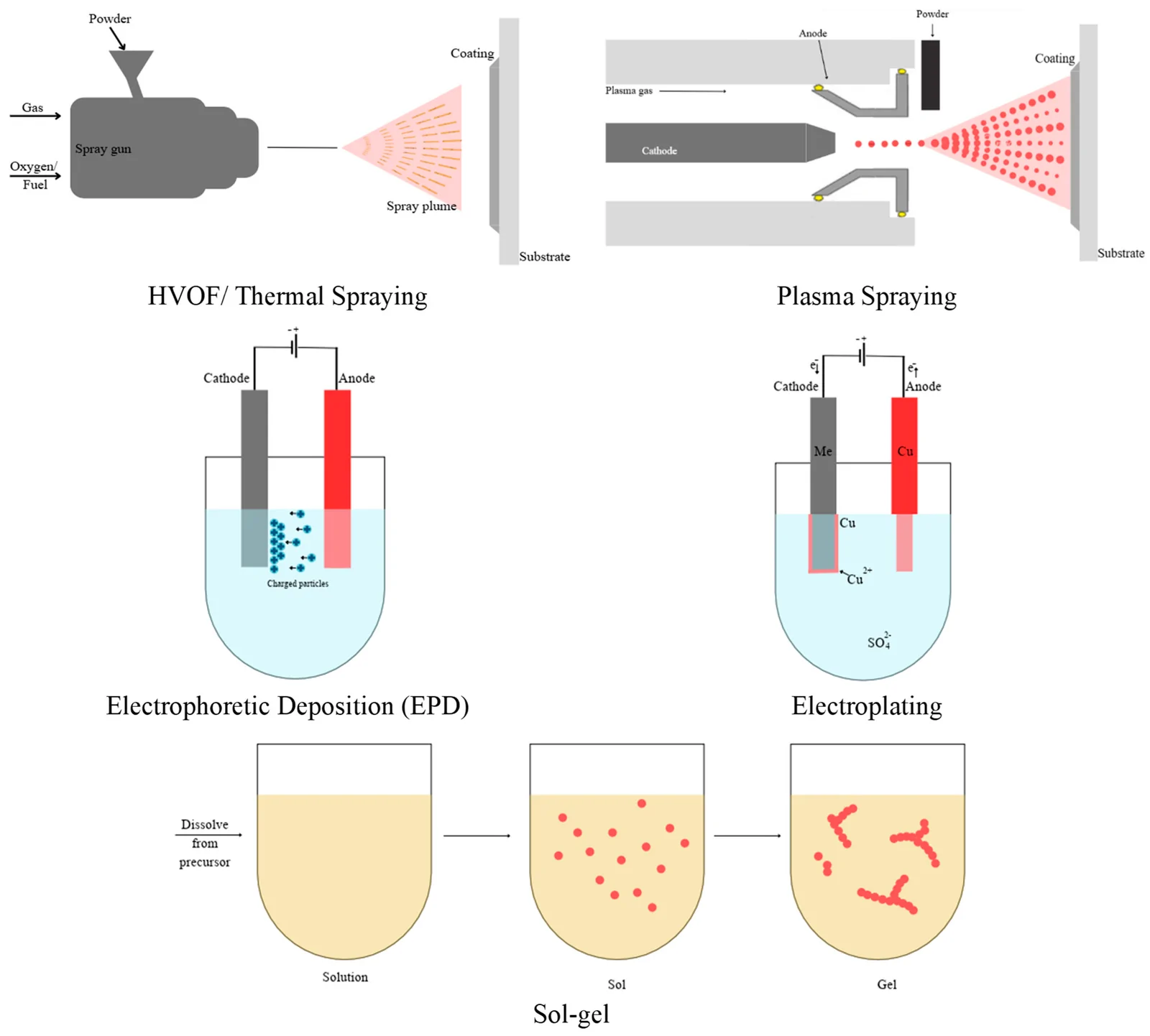
Main coating deposition techniques used for marine protective systems.

The principal coating and deposition techniques considered in this study are schematically illustrated in Figure 4, encompassing thermal spray, plasma spray, electrophoretic/electrochemical deposition, and sol–gel approaches.

Among high-energy deposition routes, high-velocity oxygen fuel (HVOF) spraying has been widely investigated because it can produce dense coatings with low porosity, high adhesion, and improved wear resistance [99].

Electrophoretic deposition (EPD) has also become relevant for corrosion protection because it enables the uniform deposition of functional materials, such as graphene oxide, onto three-dimensional metallic structures [100]. Related electrochemical routes have further demonstrated that graphene oxide/silane composite coatings can combine corrosion resistance with anti-biofouling performance through controlled surface chemistry and special wettability [101]. In parallel, electrodeposited zinc and zinc-alloy coatings remain important for marine applications because they provide sacrificial or barrier-type protection depending on alloy composition and coating structure [102].

Modern electroplating continues to evolve through improvements in bath chemistry, current distribution, additive control, and environmental management [103]. Zinc-nickel alloy coatings are particularly relevant because they can improve the corrosion resistance of mild steel while maintaining acceptable mechanical and electrochemical performance [104]. Thermal spray technologies, including post-treated Ni-based coatings, continue to be used where high wear resistance and strong surface protection are required [105]. For maritime applications exposed to cavitation or hydrodynamic loading, process selection becomes critical because coating adhesion, hardness, and microstructure strongly influence cavitation erosion resistance [106]. More broadly, thermally sprayed and thermally diffused protective coatings have been extensively reviewed as important strategies for improving the corrosion resistance of steel structures [107]. Modern surface engineering therefore focuses on producing dense, adherent, and defect-controlled coating layers capable of resisting aggressive marine exposure.

### 4.1. Thermal and Kinetic Spraying Techniques

Rather than viewing thermal spraying solely as a heat-based process, these techniques can also be classified according to the thermal and kinetic energy transferred to the particles before impact. Thermal spray methods such as high-velocity oxygen fuel (HVOF) rely on high gas velocities and controlled combustion to accelerate feedstock particles toward the substrate, where they deform, flatten, and consolidate into dense coating layers [99]. This mechanism is particularly useful for producing coatings with low porosity, high bond strength, and good wear resistance, which are essential for marine components exposed to abrasion, saltwater, and mechanical loading.

Kinetic-driven approaches, such as cold spraying, rely mainly on particle impact velocity rather than feedstock melting. This can reduce oxidation, phase transformation, and thermally induced residual stresses, making kinetic spraying attractive for temperature-sensitive materials and repair applications. In marine protection, thermally sprayed aluminum (TSA) remains particularly relevant because it provides sacrificial protection through preferential oxidation, thereby limiting the localized dissolution of the underlying steel [78]. For structures subjected to high-shear hydrodynamic forces, the selection criteria extend beyond corrosion resistance and include cavitation erosion resistance, delamination behavior, and coating adhesion under cyclic loading. In this context, process selection for cavitation-resistant coatings is especially important because spray parameters, substrate preparation, and post-treatment conditions determine the final microstructure and durability of the coating [106].

Thermal spray coatings may also require post-treatment to improve long-term marine performance. Sealing, remelting, heat treatment, or surface modification can reduce interconnected porosity, limit electrolyte penetration, and improve coating cohesion [105]. These aspects are important because pores, microcracks, and weak splat boundaries can act as preferential pathways for chloride ions and moisture, accelerating underfilm corrosion. Therefore, thermal and kinetic spraying techniques should be selected not only according to the coating material, but also according to the marine exposure zone, expected mechanical loading, repair feasibility, and required service life [105,107].

### 4.2. Electrochemical Deposition and Electroplating

Electroplating is particularly useful for components that require high dimensional accuracy, such as threaded fasteners, valves, connectors, shafts, and complex metallic parts used in maritime systems [103]. Unlike line-of-sight techniques, the electrochemical bath allows for metal ion reduction over complex geometries, provided that current distribution is managed through proper electrolyte agitation, anode positioning, additive chemistry, and bath composition [102]. This makes electroplating especially valuable for small or geometrically complex components that cannot be efficiently protected by thermal spray methods.

Zinc and zinc-alloy electrodeposits are widely used because they can provide sacrificial protection to steel substrates in chloride-containing environments [102]. However, the performance of electrodeposited coatings depends strongly on coating thickness, alloy composition, grain size, surface morphology, and the presence of defects or pores. In marine exposure, localized coating defects can accelerate galvanic interactions and promote underfilm corrosion if the coating is not sufficiently compact or well adhered.

The modern advancement in this field is centered on the optimization of cathodic efficiency and coating composition. By manipulating the electrolyte composition with organic surfactants, complexing agents, and process additives, engineers can suppress hydrogen evolution, which is a common cause of porosity and embrittlement [103]. Zinc-nickel alloy coatings are particularly important because nickel addition can improve corrosion resistance compared with pure zinc coatings, while maintaining the sacrificial character of the deposit [104]. This dual focus on corrosion inhibition and mechanical robustness supports the transition from simple metallic layers toward functional, long-lasting electrochemical surface coatings.

### 4.3. Electrophoretic Deposition (EPD) and Composite Architectures

Electrophoretic deposition (EPD) has evolved from a conventional coating application method into a useful technique for assembling nanostructured composite coatings [100]. By applying a potential difference across a stable suspension, charged particles migrate toward the electrode surface and form a coating layer. This allows the deposition of diverse particulate phases, including graphene oxide flakes, ceramic particles, hybrid organic-inorganic structures, and inhibitor-loaded carriers. In corrosion protection, graphene oxide is especially relevant because its sheet-like morphology can create tortuous diffusion pathways for water, oxygen, and chloride ions [100].

The key advantage of EPD lies in the self-limiting nature of film growth. As the deposited layer becomes thicker, its electrical resistance increases, which gradually reduces the local deposition rate and promotes a more uniform thickness even over complex three-dimensional surfaces [100]. This feature is particularly useful for marine components with complicated geometries, where conventional line-of-sight coating techniques may produce non-uniform coverage. Furthermore, EPD can be performed using aqueous or low-solvent suspensions, which supports the development of more environmentally compatible coating processes.

Recent developments also show that electrochemically deposited graphene oxide/silane composite coatings can combine barrier protection with controlled surface wettability and antibiofouling behavior [101]. Such coatings are relevant for marine applications because corrosion and biofouling often occur simultaneously, and the protective layer must reduce both electrolyte transport and biological adhesion. Therefore, EPD and related electrochemical deposition routes offer an important platform for designing multifunctional coatings that integrate barrier performance, surface functionality, and environmental compatibility.

### 4.4. Plasma Spraying

Plasma spraying is commonly used when the objective is not only corrosion resistance, but also the integration of wear-resistant ceramic phases capable of withstanding dynamic thermal cycling, erosion, and mechanical loading. The plasma plume reaches high temperatures, allowing the deposition of refractory oxides and ceramic materials that would be difficult to apply through conventional casting or low-temperature coating methods [107]. This makes plasma spraying suitable for components exposed to combined thermal and corrosive stresses, including marine engine parts, exhaust components, pumps, valves, and other high-performance metallic surfaces.

The critical challenge in plasma spraying is the management of porosity, oxidation, and residual stresses formed during the rapid quenching of molten or semi-molten droplets. If these stresses are not controlled, brittle ceramic layers may develop microcracks, delamination, or weak interfaces that reduce long-term durability. Advanced plasma spray regimes can incorporate controlled cooling, optimized stand-off distance, adjusted feedstock particle size, and post-treatment strategies to improve coating cohesion and reduce defect density [105,107]. These measures can promote a more favorable stress state within the coating and may help inhibit crack propagation typically seen in brittle ceramic layers.

For marine applications, plasma-sprayed coatings are particularly valuable when corrosion protection must be combined with resistance to wear, erosion, and thermal degradation. However, their use requires careful optimization because ceramic coatings may provide high hardness and chemical stability but can also be vulnerable to cracking if adhesion and residual stress are not properly controlled. Therefore, plasma spraying is best understood as a high-performance deposition route that must be tailored to the specific loading conditions of the marine component [105,107].

### 4.5. Sol–Gel Processing and Molecular-Level Coating Design

The sol–gel route represents a transition from mechanical deposition to chemical synthesis directly on the substrate surface. Through controlled hydrolysis and condensation reactions of metal alkoxide or organosilane precursors, hybrid organic-inorganic networks can be formed with tunable composition, porosity, adhesion, and barrier properties [108,109]. This molecular-level control is particularly valuable for corrosion protection because the coating architecture can be adjusted to reduce the transport of water, oxygen, and chloride ions toward the metallic substrate.

Hybrid sol–gel coatings are especially relevant for marine and saline environments because they can combine the advantages of inorganic networks, such as chemical stability and hardness, with the flexibility and hydrophobicity of organic components [109]. In corrosion protection applications, these coatings act primarily as compact barrier layers, but their performance can be further improved by incorporating functional additives, corrosion inhibitors, or metal oxide components. Experimental studies on hybrid organosilicon sol–gel coatings loaded with metal oxide additives have shown that such modifications can improve barrier properties and corrosion resistance in 3.5% NaCl solution, with zirconium-based additives providing particularly effective protection compared with the unmodified hybrid coating [108].

An additional advantage of sol–gel technology is its compatibility with environmentally oriented and multifunctional coating design. Because sol–gel synthesis can be performed under relatively mild conditions, it enables the incorporation of antibacterial, antifouling, hydrophobic, or inhibitor-loaded components without requiring highly aggressive processing routes [109,110]. In marine applications, this is important because surface protection must address not only electrochemical corrosion, but also biofouling and bacterial colonization. Eco-sustainable sol–gel-based materials have therefore been investigated as promising solutions for antifouling and antibacterial surface protection, including marine applications [110].

This chemical adaptability makes sol–gel layers promising candidates for protecting sensitive instrumentation, sensors, small metallic components, or high-performance marine electronics where traditional thermal or electrochemical deposition methods may be too aggressive. However, their practical performance depends strongly on precursor chemistry, curing conditions, coating thickness, crack formation, adhesion, and long-term stability under immersion or cyclic humidity exposure. Therefore, sol–gel coatings are best understood as part of hybrid protection strategies, where molecular-level design complements conventional coating systems and supports the development of multifunctional, environmentally compatible marine protective layers [108,109,110].

Substrate pre-treatment is a critical factor governing coating adhesion and long-term protective performance. Surface cleaning removes salts, oils, corrosion products, and other contaminants that can create weak interfacial regions, while mechanical roughening can increase the effective bonding area and improve mechanical anchoring. Its importance varies with the deposition route. For thermal and plasma spraying, adequate surface roughness and cleanliness are essential for particle anchoring and resistance to delamination, whereas electrochemical and electrophoretic deposition require a clean and chemically stable conductive surface to promote uniform deposition. For sol–gel systems, surface activation and interfacial compatibility are particularly important because coating adhesion depends strongly on the interaction between the deposited hybrid network and the metallic substrate. Insufficient pre-treatment can therefore promote interfacial defects, premature delamination, electrolyte penetration, and a reduction in the effective service life of the coating [103,106,107,109].

Because the final coating properties depend strongly on processing conditions, Table 2 summarizes representative operating parameters together with the main advantages and limitations of the deposition techniques discussed in this section. The reported values should be regarded as representative rather than universal, since the optimal processing window depends on the coating composition, feedstock characteristics, substrate, and equipment configuration.

## 5. Performance Evaluation Methods for Marine Protective Coatings

For a protective coating to be considered suitable for marine applications, its resistance to degradation must be demonstrated through controlled and reproducible testing procedures. The marine environment is highly aggressive because it combines salinity, moisture, temperature variation, ultraviolet radiation, mechanical wear, and biological activity [3,5,15]. Since no single laboratory test can reproduce all these degradation factors simultaneously, coating performance is usually evaluated through complementary methods, each targeting a specific failure mechanism. In active coating systems, especially those incorporating corrosion inhibitors, evaluation must also consider inhibitor type, release mechanism, electrochemical behavior, efficiency, and environmental impact [111]. Long-term studies on marine and offshore coating systems further show that laboratory testing must be correlated with real exposure data from stationary marine sites and operating vessels in order to obtain reliable durability predictions [112]. Among the available characterization methods, electrochemical impedance spectroscopy (EIS) is particularly important because it provides information on the corrosion mechanism and electrochemical response of steels exposed to corrosive environments [113]. Laboratory studies on waterborne anticorrosive paints, including polyaniline-based nanocomposite dispersions tested in 3.5% NaCl solution, also illustrate the relevance of electrochemical and saline exposure testing for evaluating protective coatings [114].

The evaluation of marine coatings must also account for coupled degradation mechanisms. Superhydrophobic coatings exposed to harsh marine conditions may experience simultaneous loss of corrosion resistance and biofouling resistance, especially when surface integrity or wettability is affected during exposure [115]. For offshore applications, corrosion and tribocorrosion testing in synthetic seawater is important because coated components can be subjected to both electrochemical attack and mechanical wear [116]. In addition, coating performance in harsh environments depends not only on the coating formulation, but also on surface preparation, application quality, inspection methods, and maintenance strategy [117]. Since some degradation processes are difficult to interpret from electrochemical data alone, in situ optical analysis can be combined with EIS in order to reduce subjectivity and link impedance changes to visible corrosion processes [118]. Reviews on organic coatings for marine applications also emphasize that protective performance depends on coating microstructure, substrate condition, environmental exposure, and the evaluation method selected [119].

Testing protocols are particularly relevant in chloride-rich environments, where corrosion represents a major operational and maintenance challenge, as also observed in seawater desalination systems [120]. A notable trend in recent years has been the development of multifactor accelerated test protocols. Instead of exposing coatings to single stressors such as salt spray alone, these protocols combine several factors in a cyclic manner, including ultraviolet radiation, salt spray, humidity, and temperature variation [121].

For cold and Arctic offshore environments, accelerated protocols may also include low-temperature phases in combination with UV and salt spray exposure, in order to better reproduce the combined stress conditions encountered in service [122]. EIS has also been used to evaluate nanofiller-modified epoxy systems, such as halloysite nanotube-filled coatings, where changes in low-frequency impedance provide information on barrier performance in 3.5% NaCl solution [123].

This chapter describes five testing methodologies commonly encountered in the literature on marine coatings. These are adhesion testing, salt spray exposure, electrochemical impedance spectroscopy, mechanical durability assessment, and accelerated weathering. For each method, the discussion covers the basic principle, the typical procedure, and the type of information that can be obtained. Where available, results from recent studies are included to illustrate typical performance differences between coating systems.

### 5.1. Adhesion Testing

The strength of the bond between a coating and its metal substrate is a primary determinant of protective performance. If adhesion is weak, water and dissolved ions can migrate along the interface, leading to blistering, underfilm corrosion, and eventually detachment of the coating [3,70]. Adhesion testing is therefore essential because even a coating with high barrier performance can fail prematurely if the coating-substrate interface is poorly prepared or chemically unstable.

The most widely used method for measuring adhesion is the pull-off test, standardized under ISO 4624:2023 [124] and ASTM D4541-22 [125]. In this test, a metal dolly is glued to the coated surface. After the adhesive has cured, a testing machine pulls the dolly perpendicular to the surface until the coating fails. The maximum force divided by the area of the dolly gives the adhesion strength in megapascals. Examination of the failed surface reveals whether failure occurred at the coating-substrate interface, within the coating itself, or within the adhesive layer [126].

Surface preparation has a strong influence on adhesion results. In this context, sandblasting followed by zinc-rich epoxy primer application and polyurethane top coating represents a practical route for improving the protection of structural steels used in marine environments [59]. Laser surface texturing has also been investigated as a method for improving adhesion strength and corrosion protection. In one study, steel surfaces patterned with V-shaped grooves using a nanosecond laser achieved higher adhesion strength than conventionally bead-blasted surfaces. The improvement was attributed to mechanical interlocking between the coating and the groove geometry, with the best results obtained when the textured area covered an optimized fraction of the total surface [126].

For conventionally grit-blasted carbon steel, epoxy coating adhesion depends on the specific epoxy formulation, the choice of hardener, the cleanliness of the surface, and the cohesion of the coating layer. Studies on epoxy-based industrial composite coatings show that both adhesion at the coating-substrate interface and cohesion within the coating are essential for long-term durability [127]. Adding functionalized nanoparticles to the coating can also improve adhesion when the particles promote interfacial compatibility or chemical bonding between the coating and the metallic substrate [59,127].

It is important to note that a coating with high initial adhesion may still lose its bond when exposed to moisture for extended periods. Water molecules can displace interfacial bonds and cause osmotic blistering. Therefore, wet adhesion testing, in which measurements are performed after immersion or humidity exposure, provides a more realistic assessment of long-term performance than dry adhesion testing alone [68,112,119].

### 5.2. Salt Spray Testing

Salt spray testing is one of the oldest and most frequently used methods for evaluating corrosion resistance. Standardized under ASTM B117-26 [128] and ISO 9227:2022 [129], the test involves placing coated samples in a closed chamber and continuously spraying them with a sodium chloride solution, commonly 5% NaCl, at a controlled temperature. The test is accelerated because the constant wetness and high salt concentration cause corrosion to develop much faster than in many natural environments [112,114].

The main advantage of salt spray testing is its simplicity and wide acceptance. It allows rapid comparison of different coating systems under identical conditions. For example, studies on anticorrosive coating systems tested in 3.5% NaCl demonstrate that saline exposure can reveal differences in barrier performance, inhibitor efficiency, and electrochemical stability between coating formulations [114]. In the case of marine and offshore coating systems, salt spray testing can also be used as part of a broader comparison with field exposure data, helping to evaluate whether accelerated laboratory degradation is representative of real service conditions [112].

Despite its popularity, salt spray testing has significant limitations. The continuous wet condition does not reproduce the cyclic wetting and drying, UV exposure, temperature variations, or biological activity that occur in real marine atmospheres. Coatings that perform well in a salt spray chamber may still fail prematurely when deployed in the field. This recognition has led to the development of cyclic test protocols, such as those described in ISO 12944-9:2018 [130], which combine salt spray with UV exposure, condensation, and low-temperature phases [112,121].

Cyclic protocols have been reported to correlate better with field exposure than continuous salt spray tests in some studies. Multifactor accelerated tests combine several environmental stressors in alternating cycles, such as UV radiation, salt spray, humidity, and temperature variation. Such protocols are useful because UV radiation can initiate surface cracking or chemical degradation, while salt-containing moisture can subsequently penetrate through coating defects and accelerate underfilm corrosion [121].

For Arctic applications, test protocols have been developed that include a freezing phase in addition to UV and salt spray. Under these conditions, epoxy-based coatings may degrade rapidly, while polyurethane coatings can maintain higher impedance and lower gloss loss throughout the test. These results indicate that coating selection for cold regions must consider the combined effects of salt, UV radiation, freezing temperature, and thermal cycling [122].

### 5.3. Electrochemical Impedance Spectroscopy (EIS)

Electrochemical impedance spectroscopy is a non-destructive technique that provides detailed information about the barrier properties of a coating and how they change over time. The method works by applying a small alternating voltage, typically between 5 and 20 mV, across a wide range of frequencies, from about 100 kHz down to 0.01 Hz. The electrical response of the coating-electrolyte system is then measured and analyzed [113].

From an electrical perspective, an intact coating behaves as a dielectric material. It has a certain capacitance, which depends on its thickness and the dielectric constant of the polymer. As water penetrates the coating, the dielectric constant increases because water has a much higher dielectric constant than dry polymer. This increase causes the measured capacitance to rise. At the same time, the formation of conductive pathways through pores or defects causes the coating resistance to decrease. When the electrolyte eventually reaches the metal surface, a new electrochemical interface appears, characterized by charge transfer resistance and double-layer capacitance. These changes can be detected by EIS long before visible corrosion becomes evident [113].

For quantitative interpretation, EIS spectra are commonly fitted using equivalent electrical circuits. For an intact coating, a simplified circuit may include the solution resistance (*R_S_*) in series with a parallel coating resistance–constant phase element branch (*R*_(*C*)_/*CPE*_(*C*)_). When electrolyte reaches the coating–metal interface, an additional charge-transfer branch (*R*_(*ct*)_/*CPE*_(*dl*)_) may be required. Constant phase elements (CPEs) are commonly used instead of ideal capacitors to account for non-ideal capacitive behavior caused by surface heterogeneity and coating defects. The fitting quality should be evaluated using residuals and goodness-of-fit parameters, while the evolution of coating resistance, CPE parameters, and charge-transfer resistance provides information on water uptake, coating degradation, and corrosion initiation [113].

A particularly useful parameter is the low-frequency impedance modulus, often evaluated at low frequencies, such as 0.01 Hz. For a high-performance coating in its initial state, this value often exceeds 10^8^ Ω·cm^2^. As the coating degrades, the low-frequency impedance drops, sometimes by several orders of magnitude. However, interpretation of EIS data can be subjective when several degradation processes occur simultaneously. For this reason, EIS can be combined with in situ optical analysis, allowing electrochemical changes to be correlated with visible surface degradation and localized corrosion processes [118].

EIS is also useful for evaluating the effect of nanofillers on coating performance. In halloysite nanotube-filled epoxy composite coatings, changes in impedance response can indicate whether nanofillers improve the barrier properties of the coating in 3.5% NaCl solution [123]. The nanotubes can create tortuous diffusion pathways that force water and ions to travel longer distances before reaching the metal substrate. However, excessive filler loading may lead to agglomeration and reduced performance, which shows that EIS is particularly valuable for identifying the optimal filler concentration in nanocomposite coatings [123].

For self-healing coatings, EIS can be used to measure the recovery of barrier properties after damage. When a coating contains microcontainers or nanocontainers loaded with corrosion inhibitors, the impedance may partially recover after scratching if active compounds are released at the damaged site. In this context, knowledge of corrosion inhibitor mechanisms, electrochemical behavior, and environmental impact is important for evaluating whether inhibitor-loaded coatings provide effective and sustainable protection [111].

### 5.4. Hardness and Abrasion Testing

Marine coatings must withstand not only chemical attack, but also mechanical degradation generated by hydrodynamic forces, suspended abrasive particles, ice contact, maintenance operations, cleaning procedures, and repeated service-related contact. In operational environments, sand and silt transported by waves can progressively abrade coating surfaces, while floating ice in polar regions may induce impact damage or surface scratching. A coating with insufficient hardness may be rapidly worn away, exposing the metallic substrate to aggressive electrolytes. Conversely, an excessively hard and brittle coating may be susceptible to cracking under impact, vibration, or thermal cycling. Therefore, mechanical durability must be evaluated together with corrosion resistance when assessing coating performance for marine applications [116].

Hardness testing provides information on the resistance of a coating to deformation, indentation, and scratching. A simple and widely used method is the pencil hardness test, standardized under ASTM D3363-22 [131], which identifies the hardest pencil grade that does not produce a permanent mark on the coating surface. A more quantitative approach is microhardness testing using a Vickers indenter, where the resistance to plastic deformation is measured under a defined load. Although these tests provide useful comparative data, hardness values should not be interpreted in isolation, but rather in relation to adhesion, flexibility, fracture behavior, and corrosion performance [116,127].

Abrasion resistance is commonly evaluated using the Taber abrasion test, standardized under ASTM D4060-25 [132]. In this method, rotating abrasive wheels are pressed against the coated surface for a defined number of cycles, after which the resulting mass loss is measured. Lower mass loss generally indicates higher resistance to mechanical wear. This test is particularly relevant for coatings applied in areas exposed to repeated mechanical stress, such as splash zones, tidal zones, decks, and components affected by suspended particles, cleaning operations, or contact wear [116].

Different coating technologies provide different balances between hardness, adhesion, flexibility, and corrosion resistance. In offshore applications, the combined action of electrochemical corrosion and mechanical wear is particularly important because coated components may experience tribocorrosion in synthetic seawater [116]. More generally, coating performance in harsh environments depends on coating selection, surface preparation, application quality, inspection procedures, and maintenance planning [117]. Consequently, no coating type can be considered universally optimal; the appropriate solution must be selected according to the specific combination of mechanical, chemical, and environmental stressors expected during service [117].

### 5.5. UV Exposure and Weathering Tests

Organic coatings are susceptible to degradation under solar radiation, especially due to the ultraviolet component of sunlight. Ultraviolet radiation has higher energy than visible light and can induce polymer chain scission, photo-oxidation, and changes in the chemical structure of the coating matrix. These processes, generally described as photodegradation, may lead to discoloration, gloss loss, chalking, surface cracking, embrittlement, and eventual loss of adhesion and barrier integrity [119].

Table 3 summarizes the main experimental methods used to evaluate the performance and durability of protective coatings, together with the corresponding properties assessed, commonly applied standards, information obtained, and key limitations.

Accelerated weathering tests are designed to simulate the effects of sunlight and moisture under controlled laboratory conditions. Commonly used standards include ASTM G154-23 [134] and ISO 16474-3:2021 [135]. These tests use fluorescent UV lamps, typically UVA-340 or UVB-313, to irradiate coated samples. Irradiation is usually combined with condensation cycles to simulate dew formation and moisture exposure. Test durations may range from hundreds to thousands of hours, depending on the performance level being evaluated. During exposure, color change, gloss retention, surface cracking, chalking, and other degradation indicators are measured at regular intervals [119,121].

Some coating types are inherently more resistant to UV degradation than others. Fluoropolymer coatings, for example, contain strong carbon–fluorine bonds and are often used in applications where long-term color and gloss stability are required. Nanoparticles such as TiO_2_ and ZnO can also improve UV resistance by absorbing ultraviolet radiation, dissipating energy, or reducing the formation of free radicals that would otherwise attack the polymer network [119]. In harsh marine conditions, degradation of surface properties can also reduce the corrosion and biofouling resistance of superhydrophobic coatings, showing that weathering tests should be interpreted together with surface stability and biological resistance [115].

The combination of UV radiation with other environmental factors can produce synergistic degradation effects. Multifactor testing has shown that UV exposure, salt spray, humidity, and temperature cycling can produce more realistic coating degradation than single-factor tests. UV radiation can initiate chemical changes and microcracking at the coating surface, while salt-containing moisture can subsequently penetrate through these defects and accelerate underfilm corrosion [121].

For Arctic environments, test protocols have been developed that include freezing phases in addition to UV and salt spray exposure. Chemical analysis of coatings subjected to such tests has shown that UV radiation and temperature cycling can promote oxidation, hydrolysis, gloss loss, and impedance reduction in organic coatings. These findings suggest that, for cold-region applications, conventional UV testing alone may be insufficient and should be replaced or complemented by combined protocols that include salt spray exposure and freezing phases [122].

Freeze–thaw resistance is also relevant for marine composite materials exposed to cold climates. Nano-gelcoat systems have been investigated for glass fiber-reinforced polymer composites used in marine applications, showing that modified gelcoat layers can influence durability under freeze–thaw exposure [133]. Based on these findings, UV and weathering tests should be interpreted together with adhesion, EIS, salt spray, freeze–thaw, and mechanical durability results to obtain a more realistic assessment of marine coating performance [119,121,122,133].

## 6. Economic and Scalability Analysis of Coating Technologies

The industrial deployment of advanced anticorrosive coating technologies is increasingly shaped by the convergence of economic constraints, scalability limitations, regulatory pressure, and sustainability-driven design requirements. Although recent advances in nanotechnology-enabled coatings, self-healing systems, superhydrophobic surfaces, and multifunctional composite architectures have significantly improved corrosion resistance, mechanical durability, and functional performance in marine environments, their transition from laboratory-scale prototypes to full-scale industrial applications remains challenging. From an industrial perspective, coating reliability depends not only on corrosion resistance, but also on adhesion, cohesion, mechanical stability, and resistance to environmental aging. Adhesion and cohesion are particularly important for epoxy-based industrial composite coatings, because interfacial failure or internal cracking can reduce long-term protective performance [127]. In marine composite applications, climatic durability is also important, as nano-gelcoat systems have been investigated for improving the freeze–thaw resistance of glass fiber-reinforced polymer composites [133].

These technical requirements are increasingly connected with broader regulatory and sustainability trends in the shipbuilding sector, where green shipbuilding strategies are shaped by current regulations, available technologies, and future environmental requirements [136].

The gap between laboratory performance and industrial implementation is primarily driven by discrepancies between performance optimization under controlled laboratory conditions and the complex operational realities of marine infrastructure, where coatings must withstand long-term electrochemical degradation, mechanical abrasion, biofouling, freeze–thaw cycling, and cyclic environmental loading. Consequently, economic feasibility, production scalability, and lifecycle sustainability emerge as interdependent evaluation dimensions that collectively determine technology readiness and market penetration potential. In particular, the integration of nanomaterials, bio-based polymers, and multifunctional coating introduces additional layers of complexity in terms of raw material supply chains, processing requirements, environmental compliance, and cost distribution across the lifecycle [136].

### 6.1. Economic Drivers of Coating Selection

The cost-effectiveness of anticorrosive coatings in marine environments must be assessed through a lifecycle-oriented framework, where total cost of ownership is determined not only by initial application expenses but also by maintenance frequency, operational downtime, and service life extension. Self-healing coating systems are economically relevant because their potential to limit localized coating damage may reduce repair frequency and maintenance interventions [137]. Similarly, nano-based smart coatings can improve corrosion mitigation through barrier enhancement, inhibitor release, and multifunctional protection mechanisms, although their economic viability depends on material cost, processing complexity, and long-term durability [138]. From a broader sustainability perspective, eco-strategies for coating development emphasize that performance improvements should be considered together with environmental impact, raw material use, and lifecycle consequences [139]. In parallel, predictive maintenance approaches in the maritime industry can support cost reduction by enabling earlier detection of degradation and better planning of inspection and repair operations [140,141,142,143,144,145].

Lifecycle economic assessment should consider not only the initial coating and application cost, but also maintenance, inspection, recoating, dry-docking, downtime, and operational expenses over the service life of the asset. Recent lifecycle studies on hull protection technologies have shown that coating-related economic performance is strongly influenced by maintenance frequency, fuel consumption, dry-docking requirements, and the expected lifetime of the protective system [146]. Similarly, life-cycle cost analysis of advanced surface coatings demonstrates that higher initial material costs may be offset by reduced maintenance requirements and longer functional lifetime [147].

A simplified lifecycle cost framework can be expressed as:

LCC = C_0_ + Σ [(C_m,t_ + C_r,t_ + C_d,t_ + C_op,t_)/(1 + r)ᵗ] (3)

where C_0_ is the initial coating and application cost, C_m,t_ represents maintenance costs, C_r,t_ recoating costs, C_d,t_ dry-docking or downtime costs, C_op,t_ operational costs, r is the discount rate, and t is the year of occurrence.

When coating alternatives generate measurable economic savings, their economic benefit can also be assessed using net present value (NPV):

NPV = −C_0_ + Σ [(S_t_ − C_t_)/(1 + r)ᵗ] (4)

where S_t_ represents the savings generated during year t and C_t_ represents the associated lifecycle costs. Positive NPVs indicate that the discounted economic benefits exceed the initial and recurring costs over the considered evaluation period.

Published lifecycle analyses provide useful quantitative benchmarks for interpreting these economic considerations. In a marine hull-protection case study, active antifouling protection was associated with a reduction in required propulsion power of up to 120 kW, and lifecycle analysis indicated economic advantages over passive protection despite the higher initial investment [146]. In another lifecycle cost study, a polyurethane-based superhydrophobic coating system was reported to have a coating material cost of approximately €3/m^2^, an initial application cost of approximately €3/m^2^, and a reapplication cost of approximately €9/m^2^, with a reported total cost benchmark of €15/m^2^ compared with €1446/m^2^ for the uncoated reference system under the assumptions of that study [147].

Although the latter analysis concerns an anti-icing rather than a marine application, it illustrates the strong influence of maintenance frequency, reapplication requirements, and operational energy demand on lifecycle economics. Therefore, such values should be interpreted as case-specific benchmarks rather than universal marine coating costs.

Conventional protective systems, particularly inorganic zinc-rich coatings and thermal-spray zinc coatings, continue to dominate many industrial applications due to their established application methodologies and compatibility with large-scale infrastructure. Comparative studies of inorganic zinc-rich coatings and thermal-spray zinc coatings show that their anticorrosion performance and predicted lifespan depend strongly on coating type, degradation mechanism, and exposure conditions [141]. However, their long-term durability under continuous chloride exposure can still lead to progressive degradation and recurring maintenance interventions, which can increase cumulative lifecycle costs in offshore and marine environments [141].

In contrast, advanced nanocomposite architectures may introduce a different cost structure, characterized by higher initial investment but potentially reduced maintenance intensity. Nano-based coatings can improve corrosion protection by increasing diffusion tortuosity, reducing electrolyte penetration, and enhancing coating functionality [138]. This results in extended inspection intervals and reduced repair frequency, which can partially offset increased material and processing costs. Superhydrophobic coatings also contribute to lifecycle cost reduction through their ability to reduce surface contamination and support self-cleaning behavior. Low-cost preparation strategies for durable, transparent, superhydrophobic coatings with good environmental stability indicate that such surfaces may become more accessible for wider industrial use, although their direct applicability to marine corrosion protection still depends on durability under real service conditions [142].

From an economic perspective, self-healing coatings may reduce preventive maintenance and repair frequency, which is particularly relevant for offshore structures where maintenance operations are logistically complex, weather-dependent, and associated with high operational costs [137,140]. Cost-effectiveness trends indicate a transition toward performance-driven lifecycle economic models, where durability, functional integration, predictive maintenance, and maintenance reduction are considered alongside initial capital expenditure considerations in coating selection strategies [139,140].

### 6.2. Scalability of Advanced Coating Technologies

Scalability remains an important limiting factor in the industrial adoption of advanced anticorrosive coatings, particularly those based on nanoscale engineering and multifunctional smart materials. While laboratory-scale research often reports improved corrosion resistance and multifunctionality, industrial-scale implementation introduces significant challenges related to process reproducibility, material stability, and manufacturing complexity. Reviews on nanotechnology for corrosion prevention show that nanomaterials can improve coating performance, but their large-scale use requires control of dispersion, interfacial compatibility, environmental safety, and process repeatability [143].

Nanotechnology-based coatings face important scalability constraints arising from nanoparticle dispersion stability, agglomeration tendencies, and interfacial incompatibilities within polymer matrices. Achieving uniform distribution of nanofillers such as graphene derivatives, silica, or metal oxides requires advanced dispersion techniques, surface functionalization strategies, and high-energy mixing processes. These requirements increase production complexity and can introduce variability in coating performance at scale if not rigorously controlled [138,143].

Bio-based and biodegradable polymer systems introduce another direction of scalability. Eco-friendly biodegradable polyurethane coatings with antibacterial and antifouling performance are relevant because they combine environmental compatibility with functional surface protection [144]. However, industrial adoption depends on the availability of raw materials, reproducible synthesis, coating stability, adhesion to substrates, and long-term performance in aggressive marine conditions. Therefore, such systems must be evaluated not only in terms of laboratory performance, but also in terms of processing cost, production consistency, and regulatory acceptance [144].

Self-healing coatings present additional scalability challenges due to the sensitivity of microencapsulated healing systems and the necessity to preserve capsule integrity throughout synthesis, storage, and application stages. Industrial scaling requires strict control over polymerization conditions, encapsulation efficiency, and mechanical stability of healing agents to ensure consistent performance under real-world operational conditions [137]. Multi-action smart coating strategies further show that active anticorrosion and self-healing systems can respond to stimuli such as pH variation, chloride concentration, moisture, and mechanical damage, but their implementation at industrial scale depends on formulation robustness and long-term reliability [145].

Superhydrophobic coatings, despite their functional advantages, face scalability limitations due to the need for hierarchical micro and nanoscale surface structuring. Replicating such structures on large and geometrically complex marine components remains technically challenging and often requires adaptation of deposition techniques that are not fully compatible with conventional industrial coating lines [142]. In contrast, zinc-rich and thermal-spray coatings demonstrate relatively high scalability due to their established industrial maturity, standardized application protocols, and compatibility with existing infrastructure. Nevertheless, under severe marine exposure conditions, these conventional systems may require hybridization with advanced functional additives or complementary protective layers to meet modern durability and sustainability requirements [141].

### 6.3. Commercial Perspectives for Marine Coating Technologies

Market adoption of anticorrosive coatings is increasingly governed by regulatory frameworks, sustainability requirements, and operational efficiency imperatives across marine and offshore industries. International regulations targeting emissions reduction, hazardous chemical elimination, and marine ecosystem protection are contributing to the transition toward environmentally compliant coating systems, including low-VOC formulations, biodegradable polymers, smart coatings, and multifunctional eco-coatings [136,139].

In the commercial shipping sector, which represents one of the major application domains for marine coatings, adoption is strongly driven by fuel efficiency optimization, hull performance enhancement, maintenance cost reduction, and environmental compliance. Life-cycle cost assessment of hull protection technologies is particularly important because coating selection can influence both operational cost and the environmental performance of vessels over time [146]. Such approaches are relevant for fishing vessels and other marine assets where hull protection technologies affect maintenance requirements, fuel consumption, and environmental friendliness [146].

The offshore energy sector, including oil, gas, and wind infrastructure, prioritizes coatings capable of maintaining structural integrity under extreme mechanical, chemical, and environmental loading conditions. Smart self-healing coatings and nano-based anticorrosive systems are receiving increasing attention due to their ability to extend service intervals and reduce maintenance interventions in remote and high-cost operational environments [137,138,145]. Biodegradable polyurethane coatings with antibacterial and antifouling functionality also represent an emerging market segment driven by environmental compliance requirements and increasing pressure to reduce marine pollution impacts.

These systems align with sustainability objectives while maintaining functional performance in corrosive and biologically active environments [144].

Superhydrophobic and anti-icing coatings are expanding into niche but high-value markets, particularly in cold-region marine infrastructure where operational safety, energy efficiency, and maintenance reduction are critical performance indicators. Environmental impact and life cycle cost analysis of superhydrophobic coatings for anti-icing applications shows that the economic and environmental performance of these systems must be evaluated together, especially when advanced surface treatments are proposed for harsh exposure conditions [147,148,149]. In addition, multifunctional SiO_2_@ZnO core–shell nanosphere coatings incorporated into polymer matrices have been investigated for sustainable marine transport, showing the potential of multifunctional coatings to combine surface protection with improved operational and environmental performance [148].

Beyond these general scale-up constraints, several technology-specific bottlenecks remain. For self-healing coatings, micro or nanocontainers must survive formulation, mixing, storage, spraying, and curing without premature rupture or release of the healing agent. The amount of active material is also finite in many extrinsic systems, which can limit repeated healing after multiple damage events. Reproducible release kinetics, compatibility between the healing agent and polymer binder, long-term storage stability, and retention of adhesion and mechanical properties after repeated wet–dry, abrasion, and UV exposure therefore remain important requirements for industrial implementation [137,145,150].

Market trends suggest a gradual shift toward multifunctional, environmentally compliant, and high-durability coating systems, although commercialization remains constrained by cost-performance trade-offs, scalability limitations, durability validation, and compatibility with existing industrial application methods [138,143,146].

### 6.4. Long-Term Environmental and Economic Implications

Sustainability-driven lifecycle cost analysis (LCCA) has become an increasingly important framework for evaluating anticorrosive coating systems, integrating economic performance metrics with environmental impact indicators such as carbon emissions, VOC release, toxicity, recyclability, maintenance frequency, and long-term ecological effects. Reviews on corrosion prevention methods for steel in marine environments emphasize that coating systems, cathodic protection, inhibitors, material selection, and surface engineering strategies must be evaluated in relation to the specific exposure conditions and service requirements of marine structures [149].

The qualitative descriptors used in Table 4 are intended to indicate relative industrial maturity and implementation characteristics based on the literature discussed in this section, rather than fixed quantitative thresholds.

Conventional zinc-based and solvent-based coatings, despite their economic advantages, may be associated with higher environmental burdens due to VOC emissions, material consumption, and repeated maintenance operations. In contrast, biodegradable polyurethane systems demonstrate improved environmental compatibility through reduced toxicity and enhanced antibacterial or antifouling functionality, aligning with increasingly stringent marine environmental regulations [144]. Nanocomposite coatings may also provide sustainability benefits through extended service life and reduced maintenance frequency, which collectively lower cumulative environmental impacts associated with repair operations and material consumption. However, uncertainties regarding nanoparticle persistence and ecological toxicity remain critical challenges requiring further investigation under evolving regulatory frameworks [143].

Ceramic-enhanced coatings and multifunctional nanocomposite systems may offer long-term sustainability advantages due to improved durability and reduced recoating frequency. Similarly, SiO_2_@ZnO multifunctional coatings contribute to sustainability by improving coating functionality and supporting more efficient marine transport systems [148]. Lifecycle assessment studies generally indicate that increasing coating durability is an effective strategy for reducing environmental footprint in marine infrastructure, primarily by minimizing maintenance-related activities such as dry-docking, transportation, and surface reapplication processes [146,147].

Additionally, predictive maintenance strategies enabled by digital monitoring and data-driven decision systems further enhance sustainability outcomes by optimizing coating performance monitoring and reducing unnecessary maintenance interventions throughout asset lifecycles [140]. Sustainability in coating technologies emerges as a multidimensional construct integrating material durability, environmental compatibility, lifecycle cost optimization, and long-term performance in marine environments, supporting the role of advanced coatings in low-carbon and resource-efficient marine infrastructure systems [136,139,146,149].

## 7. Research Perspectives and Future Developments in Marine Protective Coatings

Future research on marine protective coatings should increasingly focus on the unresolved limitations that restrict the transition of advanced systems from laboratory development to long-term naval and offshore service. Key priorities include improving long-term durability, environmental compatibility, multifunctionality, industrial scalability, and the integration of coating performance with condition-monitoring and predictive-maintenance strategies.

One important future direction is the long-term validation and industrial translation of smart self-healing coatings [150]. In parallel, sustainable polymer-based coatings are gaining importance because they can reduce environmental impact while keeping protective functionality in corrosive environments [151]. Multifunctional polymer systems are also being explored, including biomass polyurethane-based composite coatings that combine anticorrosion, radiative cooling, UV shielding and recyclability in a single material platform [152]. At the same time, durability assessment under realistic marine exposure remains essential, particularly for coatings exposed to alternating hydrostatic pressure, where water transport and resistivity through the coating thickness can influence long-term protection [153].

Bio-inspired and antifouling strategies represent another major research direction. Coral-inspired coatings and related bioinspired concepts provide models for reducing biological adhesion without relying on highly toxic biocides [154]. Low-roughness antifouling coatings are also relevant because they can reduce hydrodynamic drag, fuel consumption and associated greenhouse gas emissions during ship operation [155]. In addition, surface modification methods such as plasma treatment, etching, functionalization and nanostructuring may improve coating adhesion, wettability and integration with metallic substrates, although their industrial applicability must be shown under realistic marine conditions [157]. For naval auxiliary systems, functional polymer-based coatings with simultaneous antiscaling and anticorrosive properties are a relevant future direction, because mineral deposition, salt accumulation and localized corrosion can occur together in seawater pipelines, heat exchangers and transport units [158].

Current research indicates that the future of marine coating technologies will likely be shaped by five interconnected directions: the replacement of toxic substances with environmentally acceptable alternatives; the development of coatings with longer functional durability; the integration of self-healing, antifouling and anticorrosive mechanisms into unified architectures; the use of data-driven and digital-twin-based monitoring for predictive maintenance; and the design of scalable, recyclable and cost-efficient coating systems compatible with industrial application routes [150,151,152,155,158]. These directions are directly aligned with the broader objective of prolonging the operational lifetime of naval materials while reducing maintenance costs, environmental impact and unplanned downtime.

### 7.1. Environmental Regulation and Sustainable Coating Design

Environmental regulation is one of the main drivers reshaping the development of marine coatings. Traditional antifouling and anticorrosive formulations have often relied on compounds that are effective but environmentally problematic, including organotin compounds, heavy-metal-based additives, persistent biocides, and volatile organic compounds. The environmental damage associated with toxic antifouling agents has already driven global restrictions and bans, encouraging the development of biocide-free and bioinspired alternatives [154]. In parallel, corrosion inhibitor research has moved toward plant extracts, biopolymers, amino acids, selected low-toxicity ionic liquids and organic–inorganic hybrids in response to the need for effective but less toxic corrosion control strategies [87,90,111].

The primary difficulty is that environmental safety and protective performance are not always easy to optimize simultaneously. Toxic additives are often effective because they interfere aggressively with biological adhesion or electrochemical reactions. Replacing them with green materials requires new mechanisms of action, such as adsorption-based inhibition, hydrophilic hydration layers, foul-release surfaces, natural antifoulants, zwitterionic structures, biopolymer-based systems or biomimetic micro/nano topographies [111,154]. However, the efficiency of these alternatives may depend strongly on pH, salinity, temperature, biological species, flow conditions and exposure duration. Green inhibitors, for example, may exhibit high inhibition efficiencies under controlled conditions, but their performance can vary due to differences in natural composition, extraction method, molecular stability and adsorption behavior on the metal surface [87,90,111].

Bio-inspired antifouling strategies represent a promising environmentally compatible direction. Coral-inspired coatings, for example, use natural antifoulant effects, foul-release mechanisms, sloughing behavior, soft surface structures and fluorescence-related strategies as conceptual models for reducing organism attachment without relying on highly toxic biocides [154]. Similarly, broader biomimetic antifouling approaches use surface microstructures, hydrogel layers, amphiphilic polymers and zwitterionic systems to reduce adhesion of bacteria, algae, barnacles and other fouling organisms [159]. These strategies are particularly relevant to naval materials because fouling not only increases hydrodynamic drag and fuel consumption but also accelerates localized corrosion and promotes microbial corrosion processes.

Nevertheless, environmental compatibility must be assessed beyond the absence of conventional toxic substances. Nanoparticle-based coatings, including ZnO, TiO_2_, polyaniline nanocomposites and cellulose- or chitosan-based nanofillers, can improve corrosion resistance and antifouling performance, but their long-term release, ecotoxicity, persistence and transformation in seawater require more systematic evaluation [114,160]. In parallel, recent developments in corrosion inhibitors show that green and hybrid inhibitor systems may reduce toxicity while maintaining electrochemical protection efficiency, although their performance depends strongly on molecular structure, adsorption behavior, electrolyte composition and exposure conditions [111].

Another regulatory challenge concerns VOC reduction and the transition toward waterborne or solvent-reduced coating systems. Sustainable polymer-based coatings and waterborne anticorrosive paints are increasingly important because they reduce occupational and environmental hazards while maintaining protective performance [114,151]. The acrylate waterborne paint incorporating polyaniline/Zn/Ni nanocomposite dispersion demonstrates that water-based systems can achieve high anticorrosive efficiency in 3.5% NaCl solution when the conductive polymer nanophase is properly dispersed within the coating matrix [114]. However, for naval applications, future studies should extend such systems toward long-term immersion, cyclic mechanical loading, seawater bioactivity and field validation.

Future environmental and regulatory development should therefore focus on three priorities. First, coating formulations must be designed according to green chemistry principles, including renewable feedstocks, lower toxicity, reduced VOC emissions and safer degradation products [90,111,151]. Second, standardized ecotoxicological testing should accompany electrochemical and mechanical characterization, especially for nanomaterial-containing systems [160]. Third, regulatory approval should be integrated early in coating development, rather than treated as a final-stage requirement. This is particularly important for naval and offshore applications, where large surface areas, long exposure times and direct contact with marine ecosystems magnify the consequences of coating leaching or degradation.

### 7.2. Long-Term Durability Under Marine Service Conditions

Durability remains the central technical challenge for coatings intended to prolong the lifetime of naval materials. Marine environments accelerate coating failure through multiple coupled pathways: water uptake, ion diffusion, osmotic blistering, adhesion loss, mechanical erosion, fouling accumulation, microbial attack and underfilm corrosion. Organic coatings provide an essential barrier against water and oxygen ingress, but their protective function gradually decreases as seawater penetrates the polymer network and reaches the coating-metal interface [59,153]. Under deep-sea or fluctuating-pressure conditions, the situation becomes more complex because alternating hydrostatic pressure can accelerate electrolyte transport and modify water diffusion behavior through coating defects or pores [153].

The study of pigmented and unpigmented epoxy coatings under marine alternating hydrostatic pressure highlights an important future requirement: coating degradation should not be evaluated only by surface appearance or simple immersion time, but by internal transport behavior and resistivity profiles through the coating thickness [153].

The use of EIS-based physical models, including power-law and two-layer models, provides a more mechanistic understanding of anti-permeability and water absorption behavior. This is particularly relevant for naval structures exposed to variable pressure conditions, such as submarines, deep-sea equipment, offshore foundations and components operating between shallow and deep marine zones [153].

Nanocomposite coatings offer a route to improved durability by increasing tortuosity, reducing permeability, enhancing mechanical strength and improving adhesion between the polymer matrix and the substrate, and recent reviews further support their potential for corrosion protection of metallic materials when nanofillers are properly dispersed within the coating matrix [161]. Cellulose nanocrystals, carbon nanotubes, inorganic nanoparticles and other bio-based or inorganic nanofillers can create more complex diffusion pathways for corrosive species and improve the mechanical integrity of the coating film [160]. However, nanocomposite performance depends heavily on dispersion quality, filler–matrix compatibility, filler concentration and interfacial bonding. Poor dispersion or agglomeration may create defects that accelerate rather than suppress coating degradation. Therefore, the future of nanocomposite coatings should emphasize controlled dispersion, surface functionalization and structure–property relationships rather than simply increasing filler content [160].

Self-healing coatings represent a more advanced durability strategy because they address the fundamental problem that coating damage is difficult to avoid in service. Smart self-healing systems can detect local stimuli such as pH variation, mechanical cracking, temperature change or redox activity and respond by releasing inhibitors or restoring polymer continuity [150]. Intrinsic systems rely on reversible chemical bonds, such as dynamic covalent bonds or supramolecular interactions, whereas extrinsic systems use microcapsules, nanocapsules, vascular networks or porous carriers loaded with healing agents or corrosion inhibitors [150]. For naval applications, these systems are highly attractive because they can reduce the progression of microdefects before they evolve into underfilm corrosion or macroscopic coating failure.

However, important limitations remain. Self-healing efficiency is often demonstrated under ideal laboratory damage conditions, while real naval structures experience repeated abrasion, fouling pressure, cyclic wet–dry exposure, salt crystallization, UV radiation and mechanical deformation. Healing agents may also be exhausted after one or several damage events, especially in capsule-based systems. Intrinsic systems may allow repeated healing, but their mechanical strength, adhesion and chemical resistance must remain compatible with marine service requirements [150]. Therefore, future work should prioritize multi-cycle healing tests, field exposure validation, compatibility with industrial coating application methods and quantitative links between healing efficiency and actual service-life extension.

Multifunctional polymer coatings also represent an important direction. Biomass polyurethane-based composite coatings derived from Jatropha oil and filled with BaSO_4_/TiO_2_ nanoparticles demonstrate that anticorrosion, radiative cooling, UV shielding and recyclability can be combined in a single coating platform [152]. The reported high UV reflectance, temperature reduction under infrared irradiation, resistance to strong acid, alkali and salt immersion, and recyclability suggest that future marine coatings may provide not only corrosion protection but also thermal management and circular material value [152]. Such multifunctional behavior is particularly relevant for exposed shipboard structures, offshore equipment, deck components and naval materials subjected to both corrosive and thermal stress.

Antiscaling and anticorrosive coatings are also significant for naval and marine auxiliary systems, especially pipelines, heat exchangers and seawater transport units. The EP/iDCNTs/Zn/PVDF functional composite coating, incorporating DTPMPA-loaded carbon nanotubes, flake zinc and PVDF, reduced CaCO_3_ deposition and improved impedance relative to conventional epoxy coating [158]. This indicates that future protective systems should address combined corrosion–scaling problems rather than treating them separately. In real marine systems, scale deposition can alter local chemistry, promote under-deposit corrosion and reduce heat-transfer efficiency; therefore, coatings with simultaneous antiscaling and anticorrosion functions are highly relevant for extending the life of naval piping and seawater systems [158].

### 7.3. Antifouling Performance and Microbiologically Influenced Degradation

Biofouling remains one of the most complex and economically significant challenges in marine materials protection. The attachment of bacteria, algae, mussels, barnacles and other marine organisms increases surface roughness, hydrodynamic resistance, fuel consumption and greenhouse gas emissions, while also contributing to corrosion acceleration and biological invasion [155,159]. In naval applications, biofouling affects hull performance, maneuverability, maintenance frequency, dry-docking intervals and operational efficiency. Therefore, antifouling coatings are not merely environmental or aesthetic technologies, but essential tools for prolonging the service life and efficiency of naval materials.

The greenhouse gas reduction potential of low-roughness antifouling coatings demonstrates the direct link between coating performance and maritime decarbonization. By reducing hull roughness, antifouling coatings can decrease fuel consumption and CO_2_ emissions. In the investigated ship cases, the use of lower-roughness antifouling coatings led to substantial fuel savings and CO_2_ reductions per route, with return-on-investment periods shorter than typical dry-docking intervals [155]. This finding is important because it shows that antifouling coatings contribute simultaneously to economic efficiency, environmental performance and material lifetime extension.

Despite this potential, antifouling coating design remains constrained by environmental restrictions on toxic biocides. Traditional antifouling systems based on organotin and other toxic substances showed strong performance but caused severe ecological damage, leading to restrictions and the need for safer alternatives. Current bioinspired strategies attempt to overcome this limitation through non-toxic or low-toxicity mechanisms, including foul-release surfaces, soft interfaces, micro/nanostructured topographies, hydrogel coatings, amphiphilic surfaces and natural antifoulant analogues [154,159]. The long term durability of bioinspired antifouling coating remains a major barrier. Many surfaces that perform well in short-term laboratory assays lose effectiveness after mechanical abrasion, biofilm maturation, surface contamination or prolonged immersion. Hydrophilic and zwitterionic coatings may resist protein and bacterial adhesion, but their mechanical robustness and adhesion to metallic substrates must be improved for naval use.

Foul-release coatings may reduce organism adhesion under hydrodynamic shear, but their performance may decrease on low-speed vessels or stationary offshore structures [159]. Coral-inspired strategies offer multiple antifouling concepts, but their translation into scalable coating systems require simplification, durability testing and compatibility with industrial coating processes [154].

For biomimetic antifouling coatings, the main bottlenecks extend beyond production cost. Their functionality often depends on precisely controlled surface chemistry, micro- or nanoscale topographies, hydrogel layers, or other bioinspired surface architectures that may be difficult to reproduce consistently in large-scale applications. In addition, long-term antifouling efficiency, environmental compatibility, fabrication complexity, and the transfer of laboratory-scale concepts to practical marine applications remain important challenges. Therefore, future development should focus on improving the durability, reproducibility, and practical applicability of these bioinspired coating systems [154,159].

Bio-corrosion and microbiologically influenced corrosion further complicate the problem. Marine biofilms can create local chemical gradients, change oxygen concentration, generate acidic or sulfide-rich microenvironments and destabilize passive films on metals. Corrosion inhibitor reviews emphasize that microbial activity, chloride concentration, pH fluctuation and temperature variation strongly affect inhibitor performance in real marine environments [87,90,111]. Future coatings for naval materials should therefore integrate antifouling and anticorrosion strategies rather than treating them as independent functions. In this respect, hybrid systems combining biopolymer-based antifouling additives, corrosion inhibitors, nanostructured barriers and self-healing carriers are particularly promising [87,111,150,160].

### 7.4. Digital Monitoring, Artificial Intelligence, and Predictive Maintenance

A major future direction in the protection of naval materials is the transition from periodic inspection toward predictive and condition-based maintenance. Traditional inspection techniques, including visual inspection and manual testing, are often insufficient for detecting early-stage coating failure, subsurface degradation, blister initiation or localized corrosion under marine exposure. AI-based degradation detection provides a pathway toward automated, faster and more reliable coating assessment in offshore and naval structures [87].

AI-based smart coating degradation detection relies on computer vision, sensor fusion, time-series modeling and simulation-assisted analytics. Convolutional neural networks can be used for image-based detection of surface rust, blistering, cracking and delamination, while recurrent or time-series models such as LSTM networks can forecast degradation trends from continuous sensor signals. Sensor fusion, combining visual, acoustic, thermal and chemical information, improves reliability in offshore environments where single-sensor systems may be affected by noise, drift, limited visibility or changing environmental conditions [87].

Machine learning can also support corrosion rate prediction by linking material composition and environmental variables to degradation behavior. The corrosion rate prediction study on low-alloy steels demonstrates that feature engineering can improve model accuracy and generalization by transforming chemical composition into atomic and physical property descriptors [53]. This is particularly important for naval materials because alloy composition, salinity, temperature, exposure time and environmental parameters interact nonlinearly. Future corrosion prediction tools should therefore combine materials data, environmental monitoring, coating condition data and field inspection results into integrated predictive models [53].

Digital twin technology provides an even broader framework for structural health monitoring. Real-time digital twin frameworks developed for floating offshore wind turbines show how monitoring data, reduced-order modeling and structural response prediction can support fatigue assessment and maintenance decision-making in marine energy systems [156]. Although the reference context is offshore wind infrastructure, the methodological implications are relevant to naval structures because similar approaches can support targeted inspections, adaptive maintenance and real-time risk assessment for steel marine platforms [156].

For coatings, a future digital twin should integrate multiple layers of information: coating formulation, application parameters, environmental exposure, electrochemical response, fouling state, inspection images, sensor data, corrosion rate predictions and maintenance history. Such a model could estimate the remaining protective lifetime of the coating and optimize recoating intervals. The integration of AI-based degradation detection with digital twins could transform coatings from passive protective layers into monitored components of a larger structural health management system [87,156].

The available literature also indicates different levels of technological maturity among data-driven approaches. Machine-learning methods have been investigated for corrosion prediction and corrosion-type identification, while AI-based coating degradation detection remains largely associated with research and proof-of-concept applications [53,87]. Real-time digital twin frameworks have been demonstrated for structural health monitoring of floating offshore wind turbines [156]. Coating specific digital twins capable of predicting remaining coating life directly from long-term marine field data remain at an early stage of development. Therefore, AI, machine learning, and digital twins should currently be regarded as complementary tools for condition assessment and predictive maintenance rather than fully established coating-management technologies.

The examples identified in the reviewed literature illustrate different stages of technological development. Machine-learning-based corrosion-rate prediction for low-alloy steels represents a data-driven research implementation in which model performance was improved through optimized input features [53]. AI-based smart coating degradation detection provides a coating-focused proof-of-concept for automated identification and assessment of coating deterioration [87]. At a more advanced system level, a real-time digital twin has been demonstrated for structural health monitoring of floating offshore wind turbines, integrating monitoring data and structural-response prediction [156]. This latter example concerns structural monitoring rather than coating specific digital twin, highlighting the remaining gap between digital-twin research and direct coating-life management in marine service. Several barriers must be addressed. AI models require high-quality, labeled and diverse datasets, but real offshore degradation data are often scarce, proprietary or inconsistent. Simulation-assisted learning may help overcome data scarcity, but simulated defects must correspond to real coating failure mechanisms. Sensor drift, power supply, data synchronization, marine biofouling of sensors and communication reliability also represent practical obstacles for long-term deployment [87]. Therefore, future work should combine laboratory-generated datasets, field exposure databases, physics-based simulations and standardized annotation protocols to improve model robustness.

### 7.5. Industrial Translation and Technology Readiness of Advanced Coatings

The practical implementation of advanced coating systems in the naval industry depends not only on laboratory performance but also on scalability, reproducibility, application compatibility and maintenance integration. Many emerging technologies, including nanocomposite coatings, self-healing systems, bioinspired antifouling layers and green inhibitor formulations, remain at intermediate technology readiness levels because their performance has not yet been validated under long-term industrial marine conditions [87,150,159,160].

Scalability is especially challenging for coatings that require precise nanoscale architecture. Nanocomposite coatings need stable dispersion of fillers, controlled viscosity, strong interfacial bonding and avoidance of agglomeration [160]. Bioinspired antifouling surfaces may require micro/nanopatterning that is difficult to reproduce over large ship hull areas [159]. Self-healing coatings require stable encapsulation, controlled release and compatibility between healing agents, inhibitors and polymer binders [150]. Even when laboratory performance is strong, the transition to shipyard scale spray application, repair coating, multilayer systems and large-area curing can introduce variability.

Surface modification is another critical aspect of implementation. The performance of a coating depends strongly on surface morphology, chemical functionality and adhesion quality. Surface modification methods, including plasma treatment, etching, nanostructuring and functionalization, can improve wettability, adhesion and coating integration. Industrial adoption requires treatments that are fast, reproducible, environmentally acceptable and compatible with large metallic structures. Future research should therefore connect surface engineering studies more directly with coating durability tests under marine conditions [157].

Sustainable polymer-based coatings are promising for industrial adoption because they align environmental objectives with practical anticorrosive function. Vegetable oils, biomass polyurethanes, polysaccharides, lignin, chitosan and cellulose-based nanofillers provide routes toward lower-toxicity and renewable coating systems [151,152,160]. Nevertheless, bio-based origin alone is not sufficient. These coatings must also satisfy marine requirements for adhesion, abrasion resistance, saltwater durability, UV stability, low permeability and repairability. The reported growth in sustainable resource-based coatings indicates increasing industrial interest, but long-term performance and standardized testing remain essential [151].

Corrosion inhibitor technologies face similar translation challenges. Recent inhibitor reviews show that organic, inorganic, hybrid and green inhibitors can reach high inhibition efficiencies under selected conditions, but performance may vary strongly with substrate, electrolyte composition, pH, temperature, microbial activity and exposure time [87,90,111]. For naval applications, inhibitors must remain effective under dynamic seawater conditions and should not leach rapidly or lose adsorption stability. Future coating systems should therefore use inhibitors in controlled-release architectures, such as nanocontainers, microcapsules, porous fillers or responsive polymer networks [87,111,150].

Technology readiness should be evaluated through a staged framework. Conventional epoxy, polyurethane and zinc-containing systems already have high industrial maturity but face sustainability and long-term performance limitations. Nanocomposite and waterborne conductive polymer coatings are moving toward higher readiness because they can be integrated into existing coating platforms [114,160]. Bio-inspired antifouling surfaces, self-healing coatings and digital monitoring systems are promising but require more field validation, multi-year exposure studies and integration with existing ship maintenance workflows [150,156,159].

### 7.6. Future Research Priorities for Naval Material Protections

Future research should focus on coating systems that combine passive barrier protection, active corrosion inhibition, antifouling function, self-healing response and digital monitoring. A promising direction is not a single “universal” coating, but modular multifunctional platforms that can be adapted to specific naval applications, such as hulls, ballast tanks, offshore wind foundations, marine pipelines, propellers, deck structures and submerged sensors.

First, long-term field validation must become a priority. Many coatings are evaluated through short immersion tests, salt-spray exposure or laboratory biofouling assays. Although useful, these methods do not fully reproduce the combined effects of hydrodynamics, UV radiation, abrasion, pressure variation, microbial colonization and temperature cycling. Future studies should combine accelerated testing with real marine exposure and should report standardized parameters such as coating thickness, surface roughness, adhesion, impedance evolution, fouling coverage, mechanical degradation and repair efficiency [153,155,159].

Second, future coatings should be designed for multifunctionality without sacrificing mechanical integrity. Anticorrosive, antifouling, antiscaling, anti-icing, radiative cooling and self-healing functions should be integrated in ways that do not compromise adhesion, toughness or processability [147,152,158]. The biomass polyurethane composite coating and the antiscaling/anticorrosive polymer-based coating show that multifunctional protection is feasible, but more work is needed to evaluate how such functions interact during long-term marine exposure [152,158].

Third, sustainability assessment should be embedded in coating development. New coatings should be evaluated not only by corrosion current density, impedance modules or fouling resistance, but also by life-cycle impact, toxicity, recyclability, resource origin and maintenance reduction potential [90,111,151,152,155]. In this context, antifouling coatings with lower roughness are particularly relevant because their environmental benefit is linked not only to reduced toxicity, but also to reduced fuel consumption and greenhouse gas emissions during ship operation [155].

Fourth, AI and digital twins should be integrated with coating science. Machine learning models can improve corrosion rate prediction, while AI-based image and sensor analysis can detect degradation earlier than traditional inspection [53,156]. Digital twins can then connect coating condition with structural response, fatigue accumulation and maintenance decision-making [156]. For naval materials, this integration could support a shift from scheduled recoating to evidence-based recoating, reducing unnecessary maintenance while preventing unexpected failures.

These research priorities should be distinguished according to their stage of development. At the laboratory and fundamental-research level, emphasis should be placed on generating well-labeled coating degradation datasets, correlating electrochemical and environmental variables with observable damage, improving model interpretability, and developing coating-specific remaining-life prediction models. At the engineering-translation level, priority should shift toward durable sensor integration, standardized data acquisition, validation under long-term marine exposure, and pilot-scale testing on representative naval or offshore structures. Such pilot studies should compare AI or digital-twin-based predictions with conventional inspection records and actual maintenance outcomes before these tools can support routine coating-management decisions [87,156].

Finally, future coating research should place greater emphasis on industrial implementation. Promising materials should be tested for sprayability, repairability, curing under shipyard conditions, compatibility with primers and topcoats, scalability of raw materials, storage stability and cost. Without these practical criteria, advanced materials may remain scientifically interesting but industrially limited. The next generation of marine coatings should therefore be developed through collaboration between materials scientists, naval architects, corrosion engineers, coating manufacturers, shipyards and environmental regulators [87,156].

In summary, the future of coating technologies for extending the lifetime of naval materials lies in the convergence of sustainability, multifunctionality, predictive intelligence, and industrial scalability. Smart self-healing systems, sustainable polymers, nanocomposite barriers, bioinspired antifouling surfaces, green inhibitors, AI-based degradation detection, and digital twins each address different parts of the same problem: maintaining the integrity and performance of materials under aggressive marine exposure for longer periods, with lower environmental and economic cost. The most successful future solutions will be those that combine these directions into robust, confirmed, scalable, and application-specific protective systems for real naval applications [87,156].

## 8. Conclusions

The literature analyzed in this review confirms that the long-term durability of naval and maritime materials is governed by the combined action of electrochemical, mechanical, environmental, and biological degradation processes. Chloride-induced corrosion, biofouling, microbiologically influenced corrosion, mechanical wear, ultraviolet exposure, and environmental aging can act simultaneously and accelerate coating deterioration and substrate degradation. Therefore, effective marine protection requires an integrated approach in which coating selection and design are adapted to the specific material, exposure conditions, and dominant degradation mechanisms.

Protective coating technologies are increasingly evolving from conventional passive barriers toward multifunctional systems capable of providing complementary protective functions. Epoxy, polyurethane, metallic, ceramic, and sacrificial coatings remain relevant because of their established performance and industrial applicability, while polymer nanocomposites, self-healing coatings, inhibitor-loaded systems, and advanced antifouling surfaces offer additional possibilities for improving barrier properties, mechanical resistance, corrosion inhibition, and biofouling control. In particular, the incorporation of nanostructured fillers into polymer matrices can restrict the transport of water, oxygen, and chloride ions and can introduce additional functionalities such as improved wear resistance, hydrophobicity, UV stability, and active protection. However, these advantages strongly depend on coating formulation, filler dispersion, interfacial compatibility, surface preparation, and deposition conditions.

The reviewed literature also demonstrates that coating performance cannot be considered independently of environmental sustainability, testing methodology, and lifecycle requirements. Bio-based polymers, green corrosion inhibitors, waterborne formulations, and environmentally compatible antifouling strategies are increasingly investigated as alternatives to conventional systems containing hazardous components. At the same time, reliable assessment of advanced coatings requires the complementary use of electrochemical, mechanical, surface, and accelerated-aging methods. Laboratory testing should increasingly be correlated with long-term exposure under realistic marine conditions, since individual accelerated tests cannot fully reproduce the combined effects of salinity, temperature variations, UV radiation, hydrodynamic forces, mechanical damage, and biological activity encountered in service.

Finally, the transition from laboratory-scale performance to practical naval and maritime implementation remains one of the main challenges for advanced coating technologies. Industrial adoption requires not only high protective performance but also reproducibility, scalability, application compatibility, repairability, reasonable lifecycle costs, and long-term environmental safety. Emerging monitoring and predictive approaches may further support coating-condition assessment and maintenance planning, but their practical value depends on integration with reliable experimental and field data. Future progress should focus on multifunctional and environmentally responsible coating systems that combine durable corrosion and biofouling protection with appropriate mechanical properties, validated long-term performance, and compatibility with large-scale industrial applications.

## Figures and Tables

**Table 1:** Comparative characteristics of protective coating systems for marine applications.

Coating System	Representative Performance/Main Advantages	Main Limitations	Marine Applicability	References
Organic polymer coatings (epoxy, PU)	High barrier performance; epoxy adhesion of approximately 30–35 MPa; PU elongation at break of approximately 50–100%	Epoxy brittleness; water and ion permeation may progressively reduce barrier performance	Hulls, decks, offshore structures, dynamically loaded components	[66,67,68,69,70,71,72]
Inorganic/ceramic coatings	High hardness, chemical stability, thermal stability, and abrasion resistance	Lower flexibility than polymeric systems; brittle behavior may limit use under deformation	High-temperature and mechanically demanding marine components	[73,74,75,76,77]
Metallic/sacrificial coatings	Active galvanic protection; established industrial use; suitable for large steel structures	Susceptibility to wear, porosity, and tribocorrosion; performance depends on sealing and microstructure	Offshore steel structures and seawater-exposed components	[78,79,80,81,82,83,84]
Functional nanocomposites	Improved diffusion barrier, mechanical reinforcement, and potential antifouling or self-healing functionality	Nanofiller agglomeration, poor dispersion, and inadequate filler–matrix interfacial compatibility can create defects, increase local permeability, and reduce barrier performance; properties also depend strongly on filler concentration.	Multifunctional and advanced marine protective systems	[76,85,86,88,89]
Sustainable/bio-based coatings	Renewable components, reduced environmental impact, potential low-VOC and multifunctional formulations	Long-term marine durability, stability, and field validation remain important limitations	Environmentally oriented marine protection	[91,92,93,94,95,96,97,98]
Hybrid/multilayer systems	Combine complementary barrier, sacrificial, mechanical, and active protection mechanisms	Increased formulation, processing, and interlayer compatibility complexity	Severe marine exposure requiring multifunctional protection	[68,76,82,83,84]

**Table 2:** Representative processing parameters, advantages, and limitations of marine coating deposition techniques.

Deposition Technique	Representative Process Parameters	Main Advantages	Main Limitations	Ref.
HVOF spraying	Flame temperature approximately 1300–3000 °C; gas velocity up to approximately 2000 m/s; performance strongly influenced by fuel/oxygen ratio, particle size, and stand-off distance	Dense coatings, low porosity, high adhesion, and high wear resistance	High equipment cost; line-of-sight process; excessive thermal input may modify feedstock phases	[99]
Zn-Ni electroplating	Current density, bath temperature, pH, and electrolyte composition are critical; an optimized current density of approximately 4 A dm^−2^ has been reported for Zn-Ni deposition	Uniform coatings on complex geometries; good thickness control; sacrificial protection	Requires strict bath control; hydrogen evolution may promote porosity or embrittlement; chemical waste management is required	[103,104]
Electrophoretic deposition (EPD)	Applied voltage, deposition time, particle concentration, and suspension stability control coating thickness and uniformity	Suitable for complex geometries; good thickness control; relatively low processing temperature	Requires stable suspensions and conductive substrates; drying may produce cracking or non-uniformity	[100]
Plasma spraying	Arc power, plasma-gas flow, particle size, feed rate, and stand-off distance are critical; representative stand-off distances are generally in the order of 75–150 mm	Suitable for refractory and ceramic materials; high hardness and wear resistance	High thermal input; residual stresses, porosity, and cracking may occur if parameters are not optimized	[105,107]
Sol–gel processing	Synthesis can be performed near room temperature; hybrid systems generally require relatively mild curing conditions depending on formulation	Mild processing conditions; tunable chemistry; suitable for thin hybrid and multifunctional coatings	Sensitive to precursor chemistry and curing; shrinkage and cracking may occur; generally limited to relatively thin films	[108,109,110]

**Table 3:** Performance evaluation methods for marine protective coatings.

Test Method	Evaluated Property	Typical Standard or Approach	Information Obtained	Main Limitation	Ref.
Adhesion testing	Coating–substrate bonding	Pull-off test, ISO 4624:2023, ASTM D4541-22	Adhesion strength, failure mode, interface quality	Dry adhesion may not represent long-term wet performance	[112,119]
Salt spray testing	Resistance to chloride-rich accelerated exposure	ASTM B117-26, ISO 9227:2022	Blistering, rusting, delamination, comparative corrosion resistance	Continuous salt fog does not fully reproduce real cyclic marine exposure	[112,114,121]
Electrochemical impedance spectroscopy (EIS)	Barrier performance and electrochemical degradation	Frequency-dependent impedance measurement in electrolyte	Coating resistance, capacitance, water uptake, corrosion initiation	Interpretation can be complex when several degradation processes occur simultaneously	[113,118]
Hardness testing	Resistance to indentation and scratching	Pencil hardness, ASTM D3363-22; Vickers microhardness	Surface hardness and resistance to deformation	High hardness alone does not guarantee durability if the coating is brittle	[116,125]
Abrasion testing	Resistance to mechanical wear	Taber abrasion, ASTM D4060-25	Mass loss, wear resistance, mechanical durability	Does not fully reproduce combined corrosion and mechanical stress	[116]
UV and weathering testing	Resistance to solar radiation and climatic aging	ASTM G154-23, ISO 16474-3:2021, cyclic UV/condensation exposure	Gloss loss, color change, cracking, chalking, embrittlement	Single-factor UV tests may not represent combined salt, humidity, and temperature effects	[119,121]
Freeze–thaw testing	Durability under cold-region cyclic exposure	Cyclic freezing and thawing protocols	Cracking, adhesion loss, impedance reduction, coating stability	Mainly relevant for Arctic or cold-region applications	[122,133]

**Table 4:** Industrial and lifecycle considerations for advanced marine coating technologies.

Technology Type	Economic Considerations	Scalability Considerations	Maintenance Implications	Sustainability Considerations	Industrial Status	Ref.
Conventional epoxy/polyurethane systems	Established materials and application routes; lifecycle cost is influenced by periodic inspection and recoating.	Compatible with conventional large-scale coating procedures.	Requires periodic inspection and recoating as barrier properties deteriorate.	Environmental performance depends on solvent content, formulation, and service life.	Established industrial use	[68,70,72]
Zinc-rich and metallic sacrificial coatings	Established protection technology; lifecycle cost depends on coating consumption, sealing, repair, and service duration.	Compatible with large steel structures and established application procedures.	May require sealing, inspection, and localized repair during service.	Zinc consumption and potential environmental release should be considered.	Established industrial use	[78,80,141]
Nanocomposite coatings	Higher formulation and processing complexity may increase initial costs, while improved durability may reduce lifecycle maintenance requirements.	Scale-up depends strongly on nanofiller dispersion, agglomeration control, and interfacial compatibility.	Improved barrier and mechanical properties may extend maintenance intervals.	Potential service-life benefits must be balanced against uncertainties regarding nanoparticle persistence and ecotoxicity.	Developing toward industrial application	[138,143]
Self-healing coatings	Additional costs arise from micro/nanocontainers, healing agents, and formulation control; potential economic benefit is associated with reduced repair frequency.	Scale-up requires reproducible encapsulation, controlled release, and preservation of capsule integrity during application.	Can locally restore barrier properties and potentially reduce repair frequency.	Longer coating lifetime may reduce material consumption, provided inhibitor release and environmental impact are controlled.	Research and pilot-stage development	[137,145]
Bio-based and sustainable coatings	Economic feasibility depends on raw-material availability, synthesis route, processing consistency, and durability.	Scale-up depends on reproducible sourcing, formulation stability, and compatibility with industrial application methods.	Potential to support lower-impact maintenance strategies if long-term durability is demonstrated.	Renewable feedstocks and low-VOC formulations can reduce environmental burden.	Emerging industrial development	[144,151]
Superhydrophobic and antifouling coatings	Potential lifecycle savings may result from reduced fouling, cleaning requirements, and drag-related fuel consumption.	Large-area application remains limited by the need to reproduce micro-/nanoscale surface structures.	May reduce fouling accumulation and cleaning frequency.	Environmental benefit depends on long-term durability, non-toxicity, and reduced fuel consumption.	Limited large-scale deployment	[142,147,152,153,154,155]
AI-based monitoring and digital twins	Requires investment in sensors, data acquisition, computing infrastructure, and model development; potential savings arise from condition-based maintenance.	Implementation depends on sensor integration, data quality, communication infrastructure, and model reliability.	Supports condition monitoring, predictive maintenance, and optimization of recoating intervals.	May indirectly reduce unnecessary maintenance, material use, and downtime.	Emerging/proof-of-concept for coating-specific applications	[140,156]

## Data Availability

No new data were created or analyzed in this study. Data sharing is not applicable to this article.
